# Plasmids conferring resistance to extended-spectrum beta-lactamases including a rare IncN+IncR multireplicon carrying *bla*_CTX-M-1_ in *Escherichia coli* recovered from migrating barnacle geese ( *Branta leucopsis*)

**DOI:** 10.12688/openreseurope.13529.1

**Published:** 2021-05-04

**Authors:** Paula Kurittu, Banafsheh Khakipoor, Michael S.M. Brouwer, Annamari Heikinheimo

**Affiliations:** 1 Department of Food Hygiene and Environmental Health, Faculty of Veterinary Medicine, University of Helsinki, Helsinki, Finland; 2 Wageningen Bioveterinary Research, Lelystad, The Netherlands; 3 Laboratory and Research Division, Microbiology Unit, Finnish Food Authority, Seinäjoki, Finland

**Keywords:** Antimicrobial resistance, whole genome sequencing, extended-spectrum beta-lactamases, multidrug resistance, migratory birds, hybrid sequencing, One Health

## Abstract

**Background: **Increasing antimicrobial resistance (AMR) is a global threat and wild migratory birds may act as mediators of resistant bacteria across country borders. Our objective was to study extended-spectrum beta-lactamase (ESBL) and plasmid-encoded AmpC (pAmpC) producing *Escherichia coli* in barnacle geese using whole genome sequencing (WGS) and to identify plasmids harboring *bla* genes.

**Methods: **Barnacle geese feces (n=200) were collected during fall 2017 and spring 2018 from an urban area in Helsinki, Finland. ESBL/AmpC-producing *E. coli* were recovered from nine samples (4.5%) and isolates were subjected to WGS on both short- and long-read sequencers, enabling hybrid assembly and determination of the genomic location of *bla *genes.

**Results: **A rare multireplicon IncN+IncR was recovered from one isolate carrying *bla*_CTX-M-1_ in addition to *aadA2b*, *lnu(F)*, and *qnrS1*. Moreover, rarely detected IncY plasmids in two isolates were found to harbor multiple resistance genes in addition to the human-associated *bla*_CTX-M-15_. Poultry-associated *bla*_CMY-2_ was identified from the widely distributed IncI1 and IncK plasmids from four different isolates. One isolate harbored an IncI1 plasmid with *bla*_CTX-M-1_ and *flor*. A chromosomal point mutation in the AmpC promoter was identified in one of the isolates. WGS analysis showed isolates carried multiple resistance and virulence genes and harbored multiple different plasmid replicons in addition to *bla-*carrying plasmids.

**Conclusions: **Our findings suggest that wild migratory birds serve as a limited source of ESBL/AmpC-producing *E. coli *and may act as disseminators of the epidemic plasmid types IncI1 and IncK but also rarely detected plasmid types carrying multidrug resistance. Human and livestock-associated ESBL enzyme types were recovered from samples, suggesting a potential for interspecies transmission. WGS offers a thorough method for studying AMR from different sources and should be implemented more widely in the future for AMR surveillance and detection. Understanding plasmid epidemiology is vital for efforts to mitigate global AMR spread.

## Introduction

As antimicrobial resistance (AMR) continues to increase unevenly worldwide ( CDC, 2019; EFSA & ECDC, 2020; WHO, 2020), it is becoming increasingly urgent to study the transmission routes of resistant bacteria and mobile genetic elements harboring resistance genes. The drivers behind increasing AMR levels in different niches, including humans, animals, and the environment, have been studied ( Holmes *et al.*, 2016), but occurrence and transmission routes of resistant bacteria need to be continuously monitored to enable early mitigation efforts.

Extended-spectrum beta-lactamase (ESBL) and plasmid-encoded AmpC (pAmpC) producing bacteria, especially *Escherichia coli*, have been successfully spreading in both humans and animals ( Ewers *et al.*, 2012). Moreover, increasing numbers of carbapenemase-producing *E. coli* are worrisome ( Nordmann & Poirel, 2019). The successful spread of AMR is partly attributed to epidemic plasmids harboring resistance genes ( Carattoli, 2011).

Regarding AMR in humans, studies have indicated human-to-human contact as the main route of transmission of ESBL-producing bacteria ( Day *et al.*, 2019; Mughini-Gras *et al.*, 2019). However, resistant bacteria may also spread via animals, food, and the environment ( EFSA, 2011; Mughini-Gras *et al.*, 2019) and their role should be continuously monitored as part of a One Health approach.

When studying bacteria resistant to antibiotics in different environments, it is important to assess the true threat any finding possesses to human or animal health. Whole genome sequencing (WGS) offers an efficient method for comparative epidemiological analysis. Different species and locations may have their unique set of bacterial sequence types (STs) and typical resistance genes but overlap between human- or animal-associated resistant bacteria and plasmids has been shown ( Carattoli, 2009; Carattoli, 2011; Rozwandowicz *et al.*, 2018). Combining short-read and long-read sequence technology provides a more accurate assembly of sequence data, which is especially important when determining the presence and structure of AMR-encoding plasmids. Plasmids occurring in multiple different regions, bacterial species, and sources can be considered epidemic ( Carattoli, 2009). Certain traits, such as pilus formation and effective conjugation machinery in IncI1 and IncN type plasmids or plasmid addiction systems, may aid in the successful spread of plasmids ( Carattoli, 2009; Carattoli, 2013).

One of the drivers of AMR has been identified to be international travel ( Holmes *et al.*, 2016; Lääveri *et al.*, 2018; van der Bij & Pitout, 2012; Woerther *et al.*, 2017). As people and goods travel across country borders, so do wild animals. Migratory birds are able to travel across continents, potentially carrying resistant bacteria with them which they have picked up from anthropogenic waste sources ( Bonnedahl & Järhult, 2014; Dolejska & Papagiannitsis, 2018). Wild birds have been shown to carry and spread ESBL-producing *E. coli* effectively between individuals in a flock ( Sandegren *et al.*, 2018), emphasizing the potential rapid spread of AMR. Waterfowl, especially barnacle geese ( *Branta leucopsis*), have become a regular sight in many countries. Thousands of geese feed and defecate in densely human-populated areas, such as near housing and recreational parks ( Elmberg *et al.*, 2017). Hundreds of thousands of barnacle geese migrate over Finland each year and feed on crop fields ( SYKE, 2016). In addition to direct contact with bird feces, food-producing animals may come into contact with crops contaminated with fecal matter, as barnacle geese increasingly forage on maintained grasslands and pastures ( Jensen *et al.*, 2018).

Barnacle geese are a protected species under the European Union Birds Directive (2009/147/EC) and listed under Annex I, which has enabled them to grow exponentially in number during recent decades, reaching a total population size of 1,390,000 in the 2010s ( Fox & Leafloor, 2018). The populations are divided into three groups: east Greenland / Scotland & Ireland, Svalbard / southwest Scotland, and Russia / Germany & the Netherlands ( Jensen *et al.*, 2018). From the 1980s to 2010s, the Russia / Germany & the Netherlands population size increased by 30 times, with birds breeding now also in the Baltic and North Sea area ( Jensen *et al.*, 2018). Originally, barnacle geese only migrated through the Baltic Sea and bred in the Arctic zone, but since 1985 increasing numbers are also breeding in coastal areas of Finland and partly inland, especially the Turku archipelago region and the capital area ( SYKE, 2019; Yrjölä *et al.*, 2017). The geese included in this study were expected to belong to this latter group.

The increasing number of birds breeding in and migrating through new areas has resulted in birds being in closer contact with humans. Increased numbers of birds have also resulted in worries of contaminated recreational and drinking water, crop damage, and conflict in cities between humans and birds. Regarding AMR in birds, gulls in particular have been studied previously, and they have been found to harbor ESBL-producing bacteria frequently, although with varying prevalence between different countries ( Stedt *et al.*, 2015). The less studied geese and other waterfowl have also been shown to carry multiple pathogens ( Elmberg *et al.*, 2017), such as *Campylobacter jejuni* in barnacle geese in Finland ( Llarena *et al.*, 2015) and *Yersinia* spp. in barnacle geese in Sweden ( Niskanen *et al.*, 2003), but studies on the role of barnacle geese in AMR dissemination and potential transmission to humans or food-producing animals is limited. Barnacle geese breeding in Svalbard and wintering in the UK were found to carry intestinal *E. coli* with a high level of resistance against colistin (100%) but only a low level against ceftazidime (2%) ( Hatha *et al.*, 2013). Waterfowl in the Netherlands and Poland have been found to have different levels of resistance against amoxicillin, enrofloxacin, and tetracycline in one study ( Kuczkowski *et al.*, 2016), and the difference was speculated to be partly attributed to differences in the environment and the proximity to humans. The resistance patterns in wild birds can mirror those found in humans in different geographic locations ( Stedt *et al.*, 2015). As technologies have advanced, hybrid sequencing now offers a powerful tool for in-depth analysis of resistance genes along with plasmid replicons, virulence genes, and phylogeny between samples extracted from different sources. The goal of the present study was to study ESBL/AmpC-producing *E. coli* in barnacle geese residing in close proximity to humans, to identify resistance and virulence genes, STs, and plasmid replicons, and to conduct comparative phylogenetic analysis to identify potential epidemic plasmids.

## Methods

### Sampling

Fecal samples from barnacle geese (n=200) were collected during one day on two occasions, September 2017 (samples H1–H100) and May 2018 (samples H101–H200), in Helsinki, Finland. The sample location, a recreational park area, was the same on both occasions. During both sampling days, a flock of approximately 500 barnacle geese were present in the area. Samples were selected on the basis of collecting fresh droppings from the area, each sample being located approximately 0.5 - 2 meters from the previous sample, totaling at covering a sampling area of approximately 100 x 100 meters. Samples were aseptically collected into individual 1 l plastic bags and transported to the laboratory for further analysis within 1 h.

### Isolation and confirmation of *Escherichia coli*

From each sample, 1.0 ± 0.1 g of feces was enriched in 9 ml sterile buffered peptone water (Oxoid, Basingstoke, UK) by incubating at 37°C overnight. Subsequently, 10 μl of the pre-enrichment was streaked onto selective MacConkey agar plates (Oxoid, Basingstoke, UK) supplemented with 1 mg/l cefotaxime and incubated at 44°C for 18–22 h. One colony from each plate with bacterial growth was re-streaked onto MacConkey agar plates with 1 mg/l cefotaxime supplement and incubated at 37°C for 18–22 h. If a plate had morphologically different colonies, a representative colony from each different growth was streaked onto an agar plate.

After achieving a pure bacterial culture, the isolate was streaked onto a bovine blood agar plate and incubated at 37°C overnight for bacterial species determination with a matrix-assisted laser desorption ionization–time of flight mass spectrometry (MALDI-TOF MS) based Bruker Biotyper (Bruker Daltonics). A score value of 2.0–3.0 was considered high-confidence and was set as the criteria. All isolates identified as *E. coli* were stored at -70°C for further characterization.

### Antimicrobial susceptibility testing

To confirm ESBL, AmpC, and/or carbapenemase production, antimicrobial susceptibility testing was performed on *E. coli* isolates with the disk diffusion method ( EUCAST, 2017). Susceptibility to third-generation cephalosporins was tested with ceftazidime (10 μg) (Neo-Sensitabs, Rosco Diagnostica, Taastrup, Denmark) and cefotaxime (5 μg) (Oxoid, Basingstoke, UK), to fourth-generation cephalosporin with cefepime (30 μg), to cephamycin with cefoxitin (30 μg), and to carbapenem with meropenem (10 μg) (Neo-Sensitabs, Rosco Diagnostica, Taastrup, Denmark). Epidemiological cutoff values were used as a reference ( EUCAST, 2019). Synergism between third-generation cephalosporins and clavulanic acid was tested with a combination disk diffusion test with cefotaxime + clavulanic acid (30 μg + 10 μg) and ceftazidime + clavulanic acid (30 μg + 10 μg) (Neo-Sensitabs, Rosco Diagnostica, Taastrup, Denmark). *E. coli* ATCC 25922 was included as a quality control. In addition to resistance to third-generation cephalosporins, resistance to cephamycin and < 5 mm difference in inhibition zones in the combination disk diffusion test were used as criteria for AmpC production, whereas ESBL production was evidenced by resistance to third-generation cephalosporins and ≥ 5 mm difference in the combination disk diffusion test.

### DNA extraction and whole genome sequencing

***Short-read sequencing***. All ESBL/AmpC-producing *E. coli* isolates (n=9) were subjected to WGS with Illumina to study the presence of AMR and virulence genes and plasmid replicons, as well as to assess the multilocus ST.

Bacterial DNA was extracted and purified with the PureLink Genomic DNA Mini Kit (Cat# K182002, Invitrogen, Thermo Fischer Scientific, Carlsbad, CA, USA) according to the manufacturer’s instructions. The assessment of DNA quality was carried out using a NanoDrop ND-1000 spectrophotometer (Thermo Fischer Scientific, Wilmington, DE, USA) and DNA quantity was measured using a Qubit 2.0 fluorometer (Invitrogen, Life Technologies, Carlsbad, CA, USA). Optical density OD260/280 of 1.8–2.0 and concentration of ≥ 50 ng/μl were set as thresholds. Library preparation was performed with Illumina Nextera XT and sequencing with Illumina Novaseq 6000 (Center for Genomics and Transcriptomics, Tübingen, Germany) with paired-end reads. Samples were sequenced with 100 × coverage, 2 × 100 bp read length, and a minimal phred quality score of 30.

***Long-read sequencing***. To study the complete sequences and to identify plasmid replicons carrying *bla* genes, all ESBL/AmpC-producing *E. coli* isolates (n=9) were additionally long-read sequenced. DNA extraction and purification were performed as described above. DNA extracts from all isolates were multiplexed in a random order with either SQK-LSK 108 or SQK-LSK 109 ligation sequence kit (Oxford Nanopore Technologies, Oxford, UK), depending on the availability of the respective flow cells, as described in more detail in the following sentences. DNA extracts from four geese isolates (H11, H21, H29, and H163) were multiplexed using the SQK-LSK108 ligation sequence kit (Oxford Nanopore Technologies, Oxford, UK) according to the manufacturer’s protocol. Libraries were loaded onto FLO-MIN106D R9.4.1 MinION flow cells (Oxford Nanopore Technologies, Oxford, UK) used with the MinION Mk1B sequencing device and sequenced with MinKNOW software v19.06.8 for 48 h. For five isolates (H5, H58, H68B, H98, and H193) DNA extracts from three or two isolates at a time were multiplexed using the SQK-LSK109 ligation sequence kit (Oxford Nanopore Technologies, Oxford, UK) according to the manufacturer’s protocol. Libraries were loaded onto FLO-FLG001 R9.4.1 Flongle flow cells (Oxford Nanopore Technologies, Oxford, UK) used with the MinION Mk1B sequencing device and sequenced with MinKNOW software v19.06.8 for 20–24 h.

### Bioinformatic analyses

Nanopore FAST5 read files were basecalled using Guppy v3.4.1 (Oxford Nanopore Technologies, Oxford, UK) with FASTQ output and demultiplexed with Qcat v1.1.0 (Oxford Nanopore Technologies, Oxford, UK). Quality trimming was performed with BBDuk (BBTools v38.71, Joint Genome Institute, USA) using a QTRIM value of seven. Hybrid assembly of Illumina and Nanopore sequences was performed with Unicycler v0.4.9b ( Wick *et al.*, 2017) set at default values. Bioinformatic analyses of bacterial DNA sequences were run on a web-based service (Center for Genomic Epidemiology, DTU, Denmark). Hybrid-assembled FASTA files were uploaded to ResFinder 4.1 ( Zankari *et al.*, 2012) to determine acquired beta-lactamase resistance genes with a 90% identity threshold and minimum length of 60%. In addition, chromosomal point mutations were searched for isolate H68B with the tool. Bacterial species identification was confirmed with KmerFinder 3.1 ( Clausen *et al.*, 2018; Hasman *et al.*, 2014; Larsen *et al.*, 2014). Bacterial multilocus sequence typing (MLST) was determined with MLST 2.0 ( Larsen *et al.*, 2012) using *E. coli* scheme 1 ( Wirth *et al.*, 2006). Virulence genes for *E. coli* isolates were determined with VirulenceFinder 2.0 ( Joensen *et al.*, 2014), using an identity threshold of 90% and a minimum length of 60%. PlasmidFinder 2.1 ( Carattoli *et al.*, 2014) was used to determine plasmid replicons located in the same contigs as beta-lactamase genes using an identity threshold of 95% and a minimum length of 60%. Plasmid STs were determined for beta-lactamase harboring plasmid replicons with pMLST 2.0 ( Carattoli *et al.*, 2014).

The plasmid sequences were annotated with Prokka v1.13 ( Seemann, 2014) and manually curated with BLASTn/BLASTp. Plasmid structures were compared with previously published reference plasmids for each different replicon type identified and visualized in BRIG v0.95 ( Alikhan *et al.*, 2011). The structure of plasmid pZPK-H11 was visualized with SnapGene software (from Insightful Science, available at https://www.snapgene.com). Visualization of the plasmid structure can also be achieved with BRIG v0.95 ( Alikhan *et al.*, 2011), for example. Studied plasmids were compared using BacCompare ( Liu *et al.*, 2019) with previously published plasmids found with a BLASTn search against the National Center for Biotechnology Information (NCBI) database and the 20 best matches with available metadata for each studied incompatibility type (for IncY plasmids only six similar, previously published plasmids with available metadata were found) were used to build a core genome MLST (cgMLST) based tree with 95% occurrence for discriminatory loci. The minimum spanning tree was visualized using GrapeTree v1.5 ( Zhou *et al.*, 2018). Information on included previously published plasmids with available metadata from NCBI GenBank are provided in Table 1.

**Table 1.** Plasmids from GenBank database included for comparison with plasmids obtained in the current study.

Plasmid name (this study)	Inc group / pMLST	*bla* gene	Plasmid name (GenBank)	Query coverage (%)	Identity (%)	*bla* gene	Inc group / pMLST	Bacterial species / ST	Accession number	Country	Source	Year of isolation
pZPK-H5	IncI1/ST38 CC-3	CTX-M-1	pCOV15	96	99.79	CTX-M-1	IncI1/ST3	*E. coli*	MG648932.1	France	Healthy broiler caecal sample	2010–2012
			pH2291-112	96	99.90	CTX-M-1	IncI1/ST3	*E. coli* / ST1638	KJ484629.1	Switzerland	Healthy human	2013
			p22638	95	99.83	CTX-M-1	IncI1-Iγ/ST3	*E. coli* / ST1638	MN419437.1	Norway	Poultry feces	2016
			pEC7	95	99.87	CTX-M-1	IncI1/ST3	*E. coli* / ST196	CP053679.1	Guadeloupe / France	Rat	2013
			pEC38	92	99.90	CTX-M-1	IncI1/ST3	*E. coli* / ST349	CP053677.1	Guadeloupe / France	Human blood sample (pyelonephritis)	2013
			p08-1118	94	99.90	CTX-M-1	IncI1/ST3	*E. coli*	MH847511.1	France	Pig digestive tract	2008
			pCOV12	94	99.90	CTX-M-1	IncI1/ST3	*E. coli*	MG648914.1	France	Healthy broiler caecal sample	2010–2012
			p2305	94	99.86	CTX-M-1	IncI1/ST3	*E. coli*	MG948334.1	Switzerland	Dog	2012–2016
			p14011252	92	99.91	CTX-M-1	IncI1/ST3	*E. coli* / ST602	MK181561.1	Denmark	Chicken meat	2014
			pESBL26	92	99.91	CTX-M-1	IncI1/ST3	*E. coli*	MT230257.1	France	River	2014
pZPK-H163, pZPK-H193	IncI1/ST23 CC-2	CMY-2	pCMY-136	100	99.95	CMY-136	IncI1	*E. coli*	MG844436.1	France	Human urinary tract sample	2002–2005
			pCE1628_I1	100	99.96	CMY-2	IncI1/ST23	*E. coli* / ST457	MT468651.1	Australia	Australian silver gull	2012
			pJIE512b	100	98.75	CMY-2	IncI1/ST2	*E. coli*	HG970648.1	Australia	Human clinical sample	N/A
			p87	97	99.99	CMY-2	IncI1/ST23	*E. coli*	CP023385.1	Scotland	Dog urinary tract infection	2002
			p17437	93	98.44	CTX-M-1	IncI1-Iγ/ST3	*E. coli* / ST57	MN419430.1	Norway	Poultry feces	2016
			pIFM3804	92	98.95	CTX-M-1	IncI1/ST108	*E. coli*	KF787110.1	UK	Pig	2009
			p07-024	92	99.09	CTX-M-1	IncI1/ST3	*E. coli*	MH847502.1	France	Pig digestive tract	2006
			pCOV30	92	99.10	CTX-M-1	IncI1/ST3	*E. coli*	MG649043.1	France	Broiler (colibasillosis)	2010–2012
			pDE105	90	99.06	TEM-1	IncI1	*Shigella sonnei*	MG569891.1	Vietnam	Human fecal sample	2000
			p15076331	92	98.51	CTX-M-1	IncI1/ST3	*E. coli* / ST156	MK181558.1	Denmark	Cattle/pork meat	2015
pZPK-H21	IncK	CMY-2, TEM-32	p4809.66	94	99.85	CMY-2	IncK2	*E. coli* / ST1431	KR905389.1	Switzerland	Human urinary tract infection	2011
			pDV45	94	99.81	CMY-2	IncK2	*E. coli* / ST1564	KR905384.1	Switzerland	Poultry meat	2013
			p23C57-3	94	99.84	CMY-2	IncB/O/K/Z	*E. coli* / ST648	LC501565.1	Japan	Broiler fecal sample	2011
			p5312.29	92	99.88	CMY-2	IncK2	*E. coli* / ST131	KR905385.1	Switzerland	Human urinary tract infection	2014
			pMbl488	90	94.81	TEM-1B (also mcr-1)	IncK2	*E. coli* / ST38	KY565558.1	Switzerland	Broiler meat (imported from Germany)	2014
			pCOV9	88	99.87	CMY-2	IncB/O/K/Z	*Escherichia coli*	MG648907.1	France	Healthy broiler caecal sample	2010–2012
			pNVI1292	88	99.86	CMY-2	IncK2	*Escherichia* *coli*/ST38	KU312044.1	Norway	Broiler meat	2012
			pDV10	88	99.99	CMY-4	IncK2	*Escherichia coli*/ ST38	KR905390.1	Switzerland	Poultry meat	2013
			pMbl536	88	97.82	TEM-1B (also mcr-1)	IncK2	*Escherichia coli*/ ST226	KY689635.1	Switzerland	Broiler meat (imported from Germany)	2014
			pTMSA970	86	99.86	CMY-2	IncK2	*Escherichia coli*/ ST420	KR905388.1	Switzerland	Poultry cloacae	2012
pZPK-H58	IncK	CMY-2	p23C16-2	99	99.98	CMY-2	IncB/O/K/Z	*Escherichia coli*/ ST23	LC501559.1	Japan	Broiler fecal sample	2011
			p17C9-3	99	99.95	CMY-2	IncB/O/K/Z	*Escherichia coli*/ ST373	LC501529.1	Japan	Broiler fecal sample	2005
			p22C48-3	98	99.78	CMY-2	IncB/O/K/Z	*Escherichia coli*/ ST362	LC501547.1	Japan	Broiler fecal sample	2010
			p24C117-3	98	99.94	CMY-2	IncB/O/K/Z	*Escherichia coli*/ ST155	LC501577.1	Japan	Broiler fecal sample	2012
			p24C25-2	97	99.97	CMY-2	IncB/O/K/Z	*Escherichia coli*/ ST57	LC501570.1	Japan	Broiler fecal sample	2012
			p16C96-3	97	99.95	CMY-2	IncB/O/K/Z	*Escherichia coli*/ ST155	LC501526.1	Japan	Broiler fecal sample	2004
			p22C25-2	97	99.95	CMY-2	IncB/O/K/Z	*Escherichia coli*/ ST10	LC501544.1	Japan	Broiler fecal sample	2010
			p18C3-2	97	99.95	CMY-2	IncB/O/K/Z	*Escherichia coli*/ ST90	LC501531.1	Japan	Broiler fecal sample	2006
			p19C79-2	97	99.95	CMY-2	IncB/O/K/Z	*Escherichia coli*/ ST354	LC501535.1	Japan	Broiler fecal sample	2007
			pTMSA992	97	99.96	CMY-2	IncK2	*Escherichia coli*/ ST420	KR905387.1	Switzerland	Poultry cloacae	2012
pZPK-H29, pZPK-H98	IncY	CTX-M-15, TEM-1B	p2018K-0756	87	99.92	CTX-M-15, TEM-1B	IncY	*Salmonella* Typhi	CP044008.1	Pakistan	Human clinical sample	2018
			p60006	87	99.98	CTX-M-15	IncY	*Salmonella* Typhi	LT906492.1	Pakistan	Human blood culture	2016–2017
			pR19.2839_ 83k	86	99.92	CTX-M-15, TEM-1B	IncY	*Salmonella* Typhi	CP046430.1	Taiwan	Human blood culture	2019
			pPGR46	71	99.98	CTX-M-15, TEM-1B	IncY	*Escherichia coli*	KM023153.1	Nigeria	Human fecal sample	2011
			pEco-CTX- M-15	70	99.90	CTX-M-15, TEM-1B	IncY	*Escherichia coli*	MF510423.1	France	Human bile sample (cholangitis)	2015
			pRC960-1	61	99.88	TEM-1B	IncY	*Shigella flexneri*	KY848295.1	China	Pig fecal sample	2009
pZPK-H11	IncN+IncR/ ST1(IncN)	CTX-M-1	p100_NDM5_ IncN	60	99.97	NDM-5	IncF-IncN	*Escherichia coli/* ST167	MT199177.1	Italy	Human urine	2018
			pH1038-142	59	99.97	CTX-M-1, TEM-1	IncF-IncN/ST1	*Escherichia coli*	KJ484634.1	Switzerland	Healthy human	2013
			pC5_41608	54	99.96	CTX-M-1	IncN	*Klebsiella* *pneumoniae*/ST2748	MF953243.1	USA	Cow	2016
			pHHA45	54	99.93	CTX-M-1	IncN/ST1	*Escherichia coli*	JX065630.1	Denmark	Pig	2006
			pL2-43	54	99.96	CTX-M-1	IncN/ST1	*Escherichia coli*/ ST295	KJ484641.1	Switzerland	Lamb	2012
			pYUHAP5-2	54	99.95	TEM-1B	IncN	*Salmonella enterica* serovar London	CP060136.1	China	Slaughtered pig	2016
			pVQS1	53	99.93	TEM-1 (also qnrS1)	IncN	*Salmonella enterica* serovar Wirchow	JQ609357.1	Switzerland	Human clinical sample	2005–2009
			pRSB203	47	99.89	TEM-1	IncN	Uncultured bacteria	JN102342.1	Germany	Wastewater treatment plant effluent	N/A
			pABWA45_3	51	99.99	TEM-1B	IncN	*Escherichia coli*/ST635	CP022157.1	Switzerland	Wastewater	2016
			pKC396	55	99.53	CTX-M-65, TEM-1	IncN	*Escherichia coli*/ST131	HM138653.1	Germany	Human clinical sample	2006
			pBK31551	61	99.70	KPC-4, TEM-1	IncN	*Klebsiella* *pneumoniae*/ ST834	JX193301.1	USA	Human clinical sample (blood culture, bacteremia)	2005
			pKC394	56	99.45	CTX-M-1, TEM-1	IncN	*Escherichia coli*/ ST131	HM138652.1	Germany	Human clinical sample	2006
			pKT58A	54	99.77	(qnrS1)	IncN/ST3	*Escherichia coli*	JX065631.1	Slovakia	Wild water bird	2010
			pMUR050	53	99.60	(armA)	IncN	*Escherichia coli*	AY522431.4	Spain	Pig clinical isolate	2002
			p15ODMR	47	99.99	TEM-1B	IncN	*Escherichia coli*/ ST10	MG904997.1	Switzerland	Pig (diarrhea)	2014–2015
			pRSB206	48	99.99	TEM-1	IncN	Uncultured bacteria	JN102344.1	Germany	Wastewater treatment plant effluent	N/A
			pRSB205	47	99.99	TEM-1	IncN	Uncultured bacteria	JN102343.1	Germany	Wastewater treatment plant effluent	N/A
			plasmid IncN	48	99.78	TEM-1A	IncN	*Klebsiella * *pneumoniae*/ST258	CP027050.1	Greece	Human clinical sample (stool)	2012–2014
			pRSB201	50	99.70	TEM-1	IncN	Uncultured bacteria	JN102341.1	Germany	Wastewater treatment plant effluent	N/A
			pQNR2078	53	99.61	(qnrB19)	IncN	*Escherichia coli*	HE613857.1	Germany	Horse clinical sample (genital tract infection)	2005

Inc group = incompatibility group; pMLST = plasmid multilocus sequence type; ST = sequence type; *E. coli* = *Escherichia coli*.

### Ethics statement

The study did not include any handling or disruption of animals, and therefore ethical approval of the research was not needed.

## Results

### Phenotypic identification of ESBL/AmpC-producing *Escherichia coli*

Out of 200 samples, 98 (49%) yielded bacterial growth on MacConkey agar plates supplemented with cefotaxime (1 mg/l). Of these, 55 (56%) samples were collected in fall 2017 and 43 (44%) in spring 2018. One colony was tested from each sample by using MALDI-TOF, revealing *E. coli* from nine (4.5%) samples. Seven (78%) of these originated from fecal samples collected on the first sampling period and two (22%) from the second sampling period. All *E. coli* samples originated from fresh, wet droppings.

***Antimicrobial susceptibility testing***. All nine isolates subjected to antimicrobial susceptibility testing were resistant to third-generation cephalosporins (cefotaxime and ceftazidime) ( Table 3). According to the combination disk diffusion test, four isolates were phenotypically ESBL producers, four isolates AmpC producers, and one isolate both an ESBL and AmpC producer. Antimicrobial susceptibility testing results are shown in Table 3.

**Table 2.** Accession numbers for isolates deposited to European Nucleotide Archive project number PRJEB42655.

Isolate name	Sample primary accession	Sample secondary accession	Illumina run accession	Oxford Nanopore run accession	Plasmid sequence accession
H5	ERS5602973	SAMEA7856498	ERR5188293	ERR5190298	ERZ1738234
H11	ERS5602974	SAMEA7856499	ERR5188294	ERR5190299	ERZ1738235
H21	ERS5602975	SAMEA7856500	ERR5188295	ERR5190300	ERZ1738236
H29	ERS5602976	SAMEA7856501	ERR5188296	ERR5190301	ERZ1738237
H58	ERS5602977	SAMEA7856502	ERR5188297	ERR5190302	ERZ1738238
H68B	ERS5602978	SAMEA7856503	ERR5188298	ERR5208198	No ESBL-plasmid
H98	ERS5602979	SAMEA7856504	ERR5188299	ERR5190303	ERZ1738239
H163	ERS5602980	SAMEA7856505	ERR5188300	ERR5190304	ERZ1738240
H193	ERS5602981	SAMEA7856506	ERR5188301	ERR5190305	ERZ1738241

**Table 3.** Antimicrobial susceptibility testing ^a^ for presumptive extended-spectrum beta-lactamase (ESBL)/AmpC-producing *Escherichia coli* from barnacle geese.

Isolate	Cefotaxime (5 μg) ^a^	Ceftazidime (10 μg)	Meropenem (10 μg)	Cefoxitin (30 μg)	Cefepime (30 μg)	Cefotaxime + clavulanic acid (30 μg + 10 μg), difference in zone diameter (mm)	Ceftazidime + clavulanic acid (30 μg + 10 μg), difference in zone diameter (mm)	Phenotype ^b^
H5	R ^c^	R	S	S	R	18	7	ESBL
H11	R	R	S	S	R	20	7	ESBL
H21	R	R	S	R	S	1	4	AmpC
H29	R	R	S	S	R	21	12	ESBL
H58	R	R	S	R	S	3	3	AmpC
H68B	R	R	S	R	S	1	2	AmpC
H98	R	R	S	S	R	20	12	ESBL
H163	R	R	S	R	R	3	4	AmpC
H193	R	R	S	R	R	0	5	ESBL + AmpC

^a^ Antimicrobial susceptibility testing with disk diffusion method according to EUCAST (2017); EUCAST (2019). ^b^ Criteria for ESBL production: resistance to third-generation cephalosporins and ≥ 5 mm difference in combination disk diffusion test; criteria for AmpC production: resistance to third-generation cephalosporins and cephamycin and < 5 mm difference in inhibition zones in combination disk diffusion test. ^c^ R = phenotypically resistant; S = phenotypically susceptible.

### Whole genome sequencing and sequence analysis

WGS revealed seven different *E. coli* STs in the sequenced isolates ( Table 4). Each sequenced isolate harbored from one to six different plasmid replicons. Three isolates carried an IncI1 plasmid replicon. Multidrug resistance was found in all of the sequenced isolates. Four of the isolates harbored genes conferring resistance to seven different antibiotic classes. In addition to beta-lactams, resistance genes were detected against aminoglycoside, fluoroquinolone, macrolide, lincosamide, streptogramin B, phenicol, sulfonamide, tetracycline, and trimethoprim. No fosfomycin or rifampicin resistance genes were detected.

**Table 4.** Genomic characteristics of hybrid sequenced *Escherichia coli* isolates and plasmid replicons from barnacle geese.

Isolate ( *E. coli* MLST)	Sampling period	Number of total contigs	ESBL- plasmid (replicon; pMLST)	*bla* gene(s) on ESBL- plasmid	Other resistance genes on ESBL- plasmid	Virulence genes on ESBL- plasmid	Phenotype ^a^	Other plasmid replicons in isolate	Other resistance genes in isolate	Other virulence genes in isolate
H5 (ST359)	Fall	6	pZPK-H5 (IncI1; ST38, CC-3)	*bla*_CTX-M-1_	*floR*	*cia*	ESBL	IncFIB, IncFIC(FII), IncFII(29)	*mdf(A), aph(6)-* *Id, aph(3″)-Ib,* *sul2, dfrA*	*astA, cea, etsC, gad, hlyF, * *iroN, iss, iucC, iutA, lpfA,* *ompT, sitA, terC, traT, tsh*
H11 (ST58)	Fall	11	pZPK-H11 (IncN+IncR; IncN: ST1)	*bla*_CTX-M-1_	*qnrS1, * *aadA2b, lnu(F)*	–	ESBL	IncFIB, IncFII, IncFII(pCoo), IncQ1	*mdf(A), aph(6)-* *Id, aph(3")-Ib,* *dfrA5, sul2,* *tet(A)*	*cea, cia, cvaC, etsC, fyuA, * *gad, hlyF, iroN, irp2, iss, * *iucC, iutA, lpfA, mchF, ompT,* *sitA, terC, traT, tsh*
H21 (ST115)	Fall	9	pZPK-H21 (IncK)	*bla*_CMY-2_, *bla*_TEM-32_	–	*traT*	AmpC	Col156, Col8282, IncFIB, IncFII, IncFII	*mdf(A)*	*air, celb, chuA, cia, eilA, etsC,* * gad, hlyF, hra, iha, iss, iucC,* * iutA, kpsE, kpsMII_K5, mcbA, * *ompT, papC, sitA, terC, traT*
H29 (ST453)	Fall	3	pZPK-H29 (IncY)	*bla*_CTX-M-15_, *bla*_TEM-1B_	*qnrS1, aph(6)-* *Id, aph(3")-Ib, * *sul2, dfrA14, * *tet(A)*	–	ESBL	–	*dfrA1, mdf(A), * *aadA1, tet(A)*	*fyuA, gad, irp2, iss, kpsE,* *kpsMIII_K10, lpfA, papC, * *sitA, terC*
H58 (ST1594)	Fall	20	pZPK-H58 (IncK)	*bla*_CMY-2_	–	*traT*	AmpC	Col8282, ColpVC	*mdf(A)*	*astA, celb, gad, gad, hra,* * iha, iss, iucC, iutA, kpsE,* *kpsMII, ompT, sitA, terC*
H68B (ST3580)	Fall	3	–	Chromosomal point mutation in AmpC promoter	–	–	AmpC	IncFIA, IncFIB, IncFIC(FII), IncFII(pHN7A8)	*mdf(A), tet(A)*	*cba, cma, etsC, gad, hlyF,* * iroN, iss, iucC, iutA, lpfA,* *ompT, sitA, terC, traT, tsh*
H98 (ST453)	Fall	3	pZPK-H98 (IncY)	*bla*_CTX-M-15_, *bla*_TEM-1B_	*qnrS1, aph(6)-* *Id, aph(3")-Ib, * *sul2, dfrA14, * *tet(A)*	–	ESBL	–	*dfrA1, mdf(A), * *aadA1, tet(A)*	*fyuA, gad, irp2, iss, kpsE, * *kpsMIII_K10, lpfA, papC, * *sitA, terC*
H163 (ST2178)	Spring	9	pZPK-H163 (IncI1; ST23, CC-2)	*bla*_CMY-2_	–	*cia*	AmpC	ColpVC, IncFIB, IncFII	*mdf(A)*	*cif, eae, espA, espF, espJ,* *gad, iss, lpfA, nleB, sepA,* * terC, tir*
H193 (ST2178)	Spring	4	pZPK-H193 (IncI1; ST23, CC-2)	*bla*_CMY-2_	–	*cia*	AmpC+ESBL	ColpVC, IncFIB, IncFII	*mdf(A)*	*cif, eae, espA, espF, espJ,* * gad, iss, lpfA, nleB, sepA, * *terC, tir*

CC = clonal complex; ESBL = extended-spectrum beta-lactamase; MLST = multilocus sequence type; pMLST = plasmid multilocus sequence type. ^a^ Based on phenotypic tests.

Human-associated resistance gene *bla*_CTX-M-15_ was found in two isolates sharing the same sequence type ST453 and plasmid replicon IncY. Additionally, four and two isolates harbored *bla*_CMY-2_ and *bla*_CTX-M-1_, respectively, which are commonly found from poultry ( EFSA, 2011; Rozwandowicz *et al.*, 2018). Interestingly, no beta-lactamase genes were recovered from isolate H68B, although this isolate was phenotypically an AmpC producer. The phenotype correlated with the genotype in all but two isolates—the aforementioned isolate H68B and isolate H193—which was phenotypically both an AmpC and an ESBL producer, but only *bla*_CMY-2_ was recovered from the WGS analysis. In addition, isolate H21 was phenotypically an AmpC producer but harbored both *bla*_CMY-2_ and *bla*_TEM-32_.

The isolates harbored a wide variety of different virulence genes with 38 different genes found altogether. The increased serum survival gene *iss*, tellurium ion resistance gene *terC*, and glutamate decarboxylase gene *gad* were found in all isolates, and the long polar fimbriae gene *lpfA* in seven isolates. Enterobactin siderophore receptor gene *iroN* and temperature sensitive hemagglutinin gene *tsh* were both found in three isolates. Two of the isolates harbored the adhesin intimin coding *eae* gene, which is associated with enteropathogenic *E. coli* ( Frankel *et al.*, 1998; Müller *et al.*, 2016), but no Shiga toxin coding genes were found.

***Plasmid analysis***. Eight out of the nine hybrid sequenced *E. coli* isolates were found to harbor a plasmid replicon with a *bla* gene. All hybrid-assembled *bla*-carrying plasmids were found to be in one circular contig.

One isolate, H68B, did not carry any plasmids with *bla* genes, but instead a chromosomal point mutation in the AmpC promoter was identified. This isolate did, however, harbor multiple IncF type replicons and resistance genes *mdf(A)* and *tet(A)* ( Table 4).

IncI1 type plasmids

In three isolates, an IncI1 type replicon harboring either *bla*_CMY-2_ (H163 and H193) or *bla*_CTX-M-1_ (H5) was identified (plasmids pZPK-H5, pZPK-H163, and pZPK-H193). Plasmid pZPK-H5 from isolate H5 was 98.7 kb in size with a G+C content of 50.2% and 112 predicted coding sequences (CDSs). Plasmid multilocus sequence typing (pMLST) analysis indicated that the plasmid belonged to ST38 CC-3. In addition to *bla*_CTX-M-1_, pZPK-H5 harbored the florfenicol resistance gene *floR*. The only virulence gene identified on this plasmid was the channel-forming colicin *cia*.

pZPK-H163 from isolate H163 and pZPK-H193 from isolate H193 were found to be identical with pairwise alignment with BLASTn. Both plasmids were 89.6 kb in size with a G+C content of 50.31% and predicted 101 CDSs. pMLST analysis identified the plasmids as ST23 CC-2. In addition to *bla*_CMY-2_, the plasmids carried the macrolide-associated resistance gene *mdf(A)*. Similar to pZPK-H5, the only virulence gene identified on these plasmids was *cia*.

**Figure 1.**
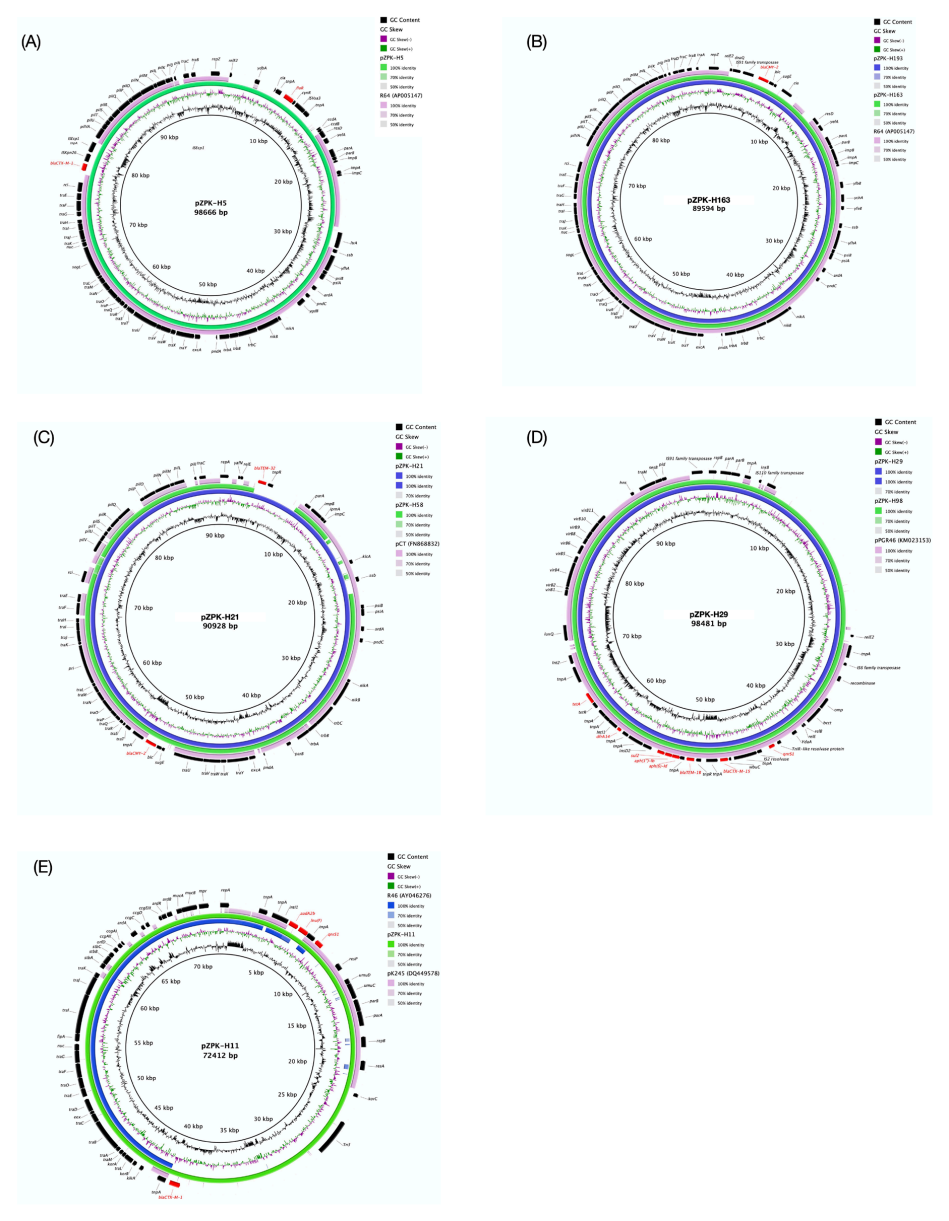
Circular comparison of the studied plasmids with previously published reference plasmids. GC content and GC skew are depicted in the inner map with distance scale. Predicted coding sequences of the plasmid named within the circle are depicted in the outer ring with antimicrobial resistance genes highlighted in red. ( **A**) pZPK-H5 compared with IncI1 type reference R64 (GenBank accession: AP005147), ( **B**) pZPK-H163 and pZPK-H193 compared with IncI1 type reference R64 (GenBank accession: AP005147), ( **C**) pZPK-H21 and pZPK-H58 compared with IncK type reference pCT (GenBank accession: FN868832), ( **D**) pZPK-H29 and pZPK-98 compared with IncY type reference pPGR46 (GenBank accession: KM023153), ( **E**) pZPK-H11 compared with IncN type R46 (GenBank accession: AY046276) and IncR type pK245 (GenBank accession: DQ449578).

Pairwise alignment with BLASTn against IncI1 type reference plasmid R64 (GenBank accession: AP005147) demonstrated high similarity between R64 and pZPK-H163 and pZPK-H193, with 89% coverage and 97.57% identity. BLASTn alignment between R64 and pZPK-H5 indicated less similarity, with 79% coverage and 98.52% identity. Alignments of the studied plasmids with the reference R64 are visualized in Figure 1A for pZPK-H5 and Figure 1B for PZPK-H163 and pZPK-H193.

The studied IncI1 plasmids demonstrated typical IncI plasmid backbones with maintenance and stability-related regions with *parAB*, *impCAB*, *ssb*, *psiAB*, *ardA*, and *pndAC* genes, and transfer-associated, shufflon, and pilus formation regions ( Figure 1A–B). In pZPK-H5, *flor* and *cia* genes were located in the accessory module, whereas *bla*_CTX-M-1_ was integrated into the shufflon region. As previously described, *bla*_CTX-M-1_ was integrated into shufflon segment B by a copy of ISEcp1, although in pZPK-H5 this IS element contains an integration of ISKpn26 ( Brouwer *et al.*, 2015). In pZPK-H163 and pZPK-H193, *bla*_CMY-2_ was located in the accessory module after the replication region.

**Figure 2.**
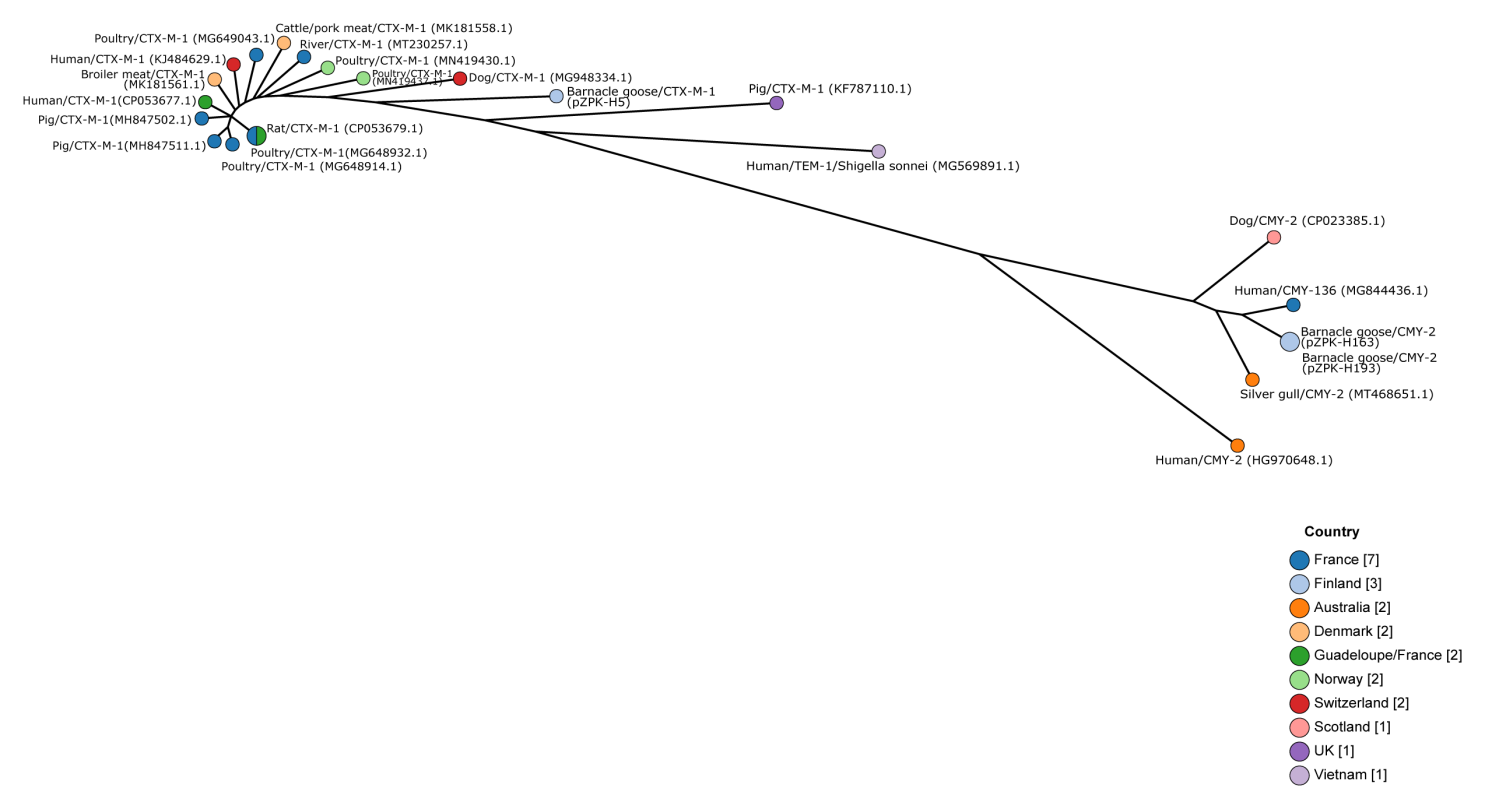
Minimum spanning tree based on core genome multilocus sequence typing of IncI1 type plasmids. Plasmids pZPK-H5, pZPK-H163, and pZPK-H193 compared with 20 previously published IncI1 plasmids from GenBank. Nodes are colored by the country of origin and node text includes information on the source, *bla* gene, and bacterial species if other than *Escherichia coli*. GenBank accession numbers are provided in parentheses.

Comparison of the studied IncI1 plasmids with the most closely related previously published plasmids from GenBank using BLASTn demonstrated that pZPK-H163 and pZPK-H193 were similar with a *bla*_CMY-136_-harboring IncI1 plasmid obtained from a human urinary tract sample in France with 100% coverage and 99.95% identity (GenBank accession: MG844436.1), a *bla*_CMY-2_-harboring IncI1 ST23 plasmid from an Australian silver gull with 100% coverage and 99.96% identity (GenBank accession: MT468651.1), and a *bla*_CMY-2_-harboring IncI1 ST2 plasmid recovered from a human clinical sample in Australia with 100% coverage and 98.75% identity (GenBank accession: HG970648.1). pZPK-H5 was found to be most similar, with *bla*_CTX-M-1_-harboring IncI1 ST3 plasmids, such as a plasmid obtained from a poultry sample in France (GenBank accession: MG648932.1) with 96% coverage and 99.79% identity, and a plasmid recovered from *E. coli* from a healthy human in Switzerland (GenBank accession: KJ484629.1) with 96% coverage and 99.90% identity. A cgMLST-based minimum spanning tree built with 75 discriminatory loci (95% occurrence) is visualized in Figure 2.

IncK type plasmids

Two isolates, H21 and H58, harbored IncK type replicons with *bla*_CMY-2_. Plasmid pZPK-H21 from isolate H21 was 90.9 kb in size with a G+C content of 52.51% and 108 CDSs. In addition to *bla*_CMY-2_, pZPK-H21 carried *bla*_TEM-32_. pZPK-H58 was 79.2 kb in size with a G+C content of 52.16% and 92 predicted CDSs. From both pZPK-H21 and pZPK-H58, only the virulence gene *traT* was identified. Pairwise alignment with BLASTn showed 88% coverage and 99.99% identity between pZPK-H21 and pZPK-H58.

Similar to IncI1 plasmids, pZPK-H21 and pZPK-H58 were found to have a typical I-complex plasmid backbone structure with maintenance and stability, conjugation, shufflon, and pilus formation regions ( Figure 1C). *bla*_TEM-32_ in pZPK-H21 was located in the accessory module, whereas *bla*_CMY-2_ in both pZPK-H21 and pZPK-H58 was located near a transfer-associated region next to *tnpA*. BLASTn pairwise alignment with IncK type reference plasmid pCT (GenBank accession: FN868832) indicated that both pZPK-H21 and pZPK-H58 shared 92.42% identity with pCT, whereas pZPK-H21 shared 81% coverage and pZPK-H58 82% coverage.

**Figure 3.**
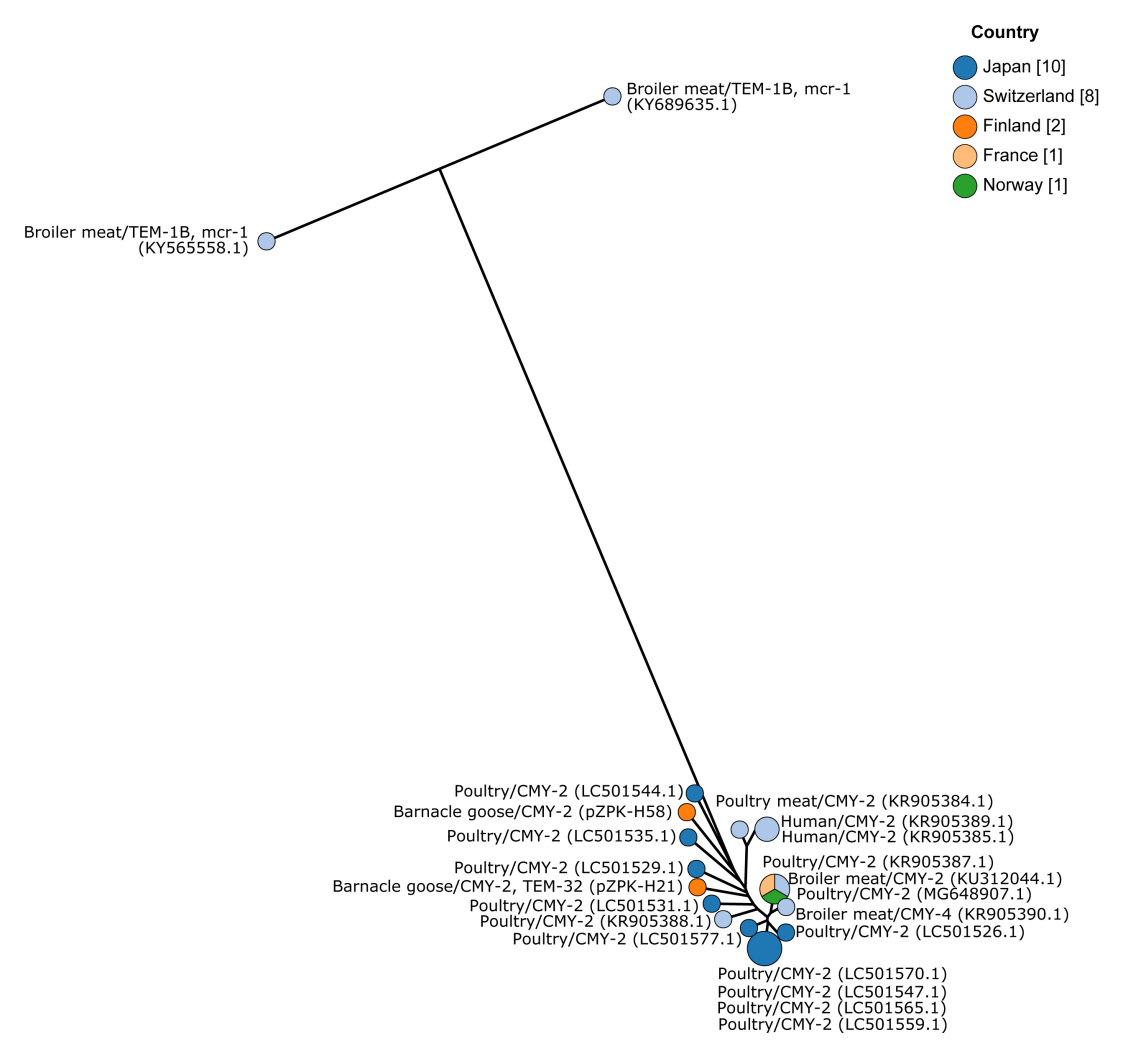
Minimum spanning tree based on core genome multilocus sequence typing of IncK type plasmids. Plasmids pZPK-H21 and pZPK-H58 compared with 20 previously published IncK or IncB/O/K/Z plasmids from GenBank. Nodes are colored by the country of origin and node text includes information on the source, *bla* gene, and possible additional *mcr-1*. GenBank accession numbers are provided in parentheses.

A BLASTn search against the NCBI database indicated that the most similar previously published plasmids consisted of poultry-associated IncK2 or IncB/O/K/Z replicons harboring *bla*_CMY-2_ ( Table 1). pZPK-H21 was also found to be similar with two IncK2 plasmids derived from *E. coli* from a human urinary tract infection in Switzerland, with 94% coverage and 99.81% identity with plasmid pDV45 (GenBank accession: KR905384.1) and 92% coverage and 99.88% identity with plasmid p5312.29 (GenBank accession: KR905385.1). A cgMLST-based minimum spanning tree was built with 64 discriminatory loci (95% occurrence) and is visualized in Figure 3.

IncY type plasmids

**Figure 4.**
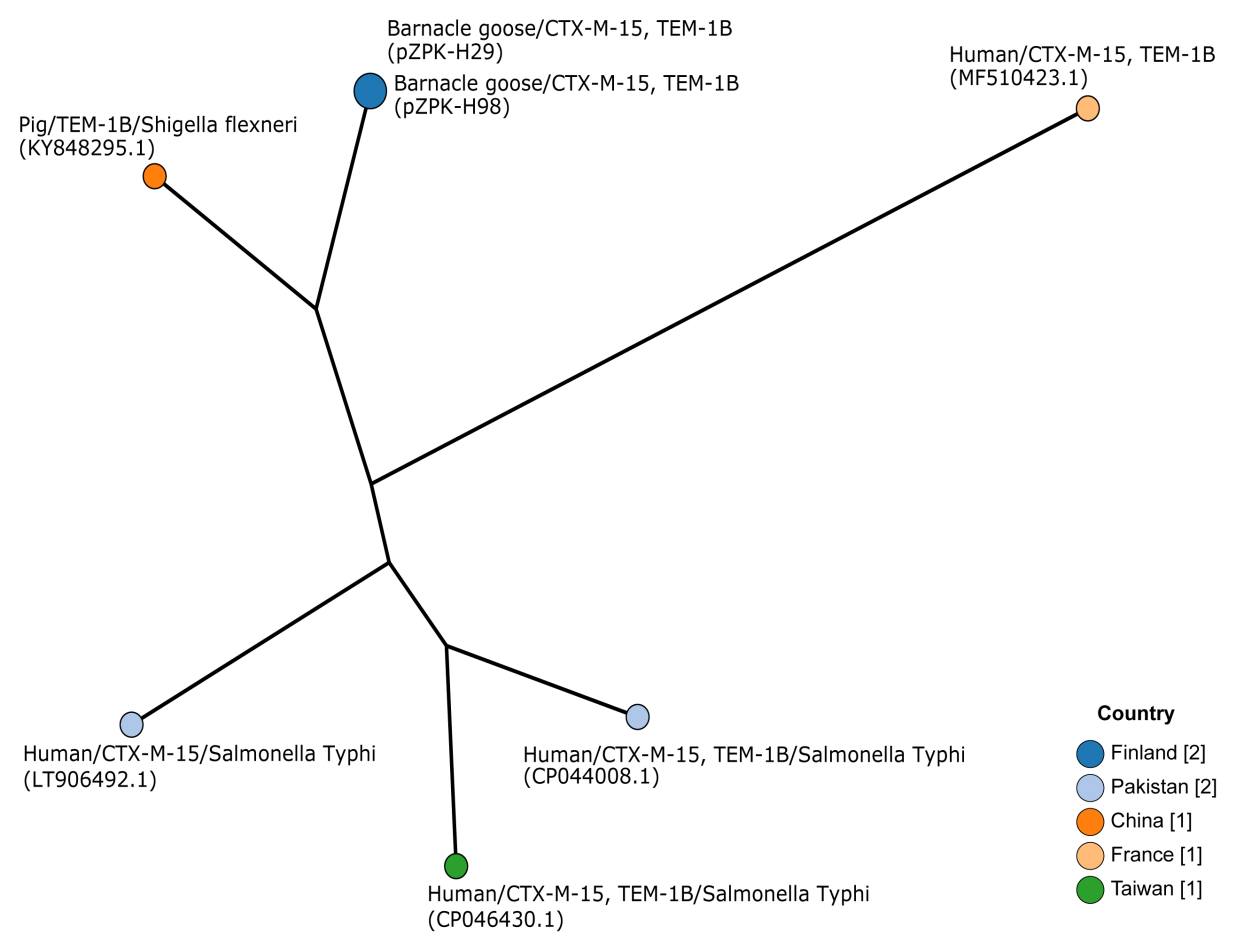
Minimum spanning tree based on core genome multilocus sequence typing of IncY type plasmids. Plasmids pZPK-H29 and pZPK-H98 compared with six previously published IncY plasmids from GenBank. Nodes are colored by the country of origin and node text includes information on the source, *bla* gene, and bacterial species if other than *Escherichia coli*. GenBank accession numbers are provided in parentheses.

Two isolates, H29 and H98, harbored IncY type replicons. pZPK-H29 from isolate H29 and pZPK-H98 from isolate H98 were highly similar when compared with pairwise alignment with BLASTn, resulting in 100% coverage and 99.94% identity. Both plasmids harbored *bla*_CTX-M-15_ and *bla*_TEM-1B_. In addition to *bla* genes, both plasmids carried the resistance genes *qnrS1*, *aph(6)-Id*, *aph(3")-Ib*, *sul2*, *dfrA14*, and *tet(A)*. Both plasmids were 98.5 kb in size, had a G+C content of 51.13% and 98 predicted CDSs in pZPK-H29, and had a G+C content of 51.12% and 99 predicted CDSs in pZPK-H98. These IncY plasmids were not found to harbor any virulence genes. BLASTn comparison with previously described IncY type plasmid pPGR46 (GenBank accession: KM023153) demonstrated 71% coverage with both pZPK-H29 and pZPK-H98, and 99.98% identity with pZPK-H29 and 99.94% identity with pZPK-H98 ( Figure 1D). A BLASTn search against the NCBI database identified only six IncY type previously published plasmids with available metadata to be similar to pZPK-H29 and pZPK-H98 ( Table 1). Three of the similar previously published plasmids were obtained from human clinical samples isolated from *Salmonella Typhi* (GenBank accessions: CP044008.1, LT906492.1, and CP046430.1) and two from *E. coli* also of human clinical sample origin (GenBank accessions: KM023153.1 and MF510423.1), all harboring *bla*_CTX-M-15_. One IncY plasmid originated from *Shigella flexneri* from a pig fecal sample harboring *bla*_TEM-1B_ (GenBank accession: KY848295.1). Phylogenetic comparison with these six previously published IncY plasmids with a cgMLST-based minimum spanning tree included 20 discriminatory loci (95% occurrence) and did not indicate clusters ( Figure 4).

IncN+IncR multireplicon

From isolate H11, two plasmid replicons, IncN and IncR, were identified on the same contig. This multireplicon plasmid pZPK-H11 was 72.4 kb in size, with a G+C content of 50.91% and 78 predicted CDSs. Pairwise alignment with BLASTn indicated that the plasmid aligned with IncN reference plasmid R46 (GenBank accession: AY046276) with a coverage of 55% and 99.73% identity, whereas alignment with previously published IncR type plasmid pK245 (GenBank accession: DQ449578) resulted in a coverage of 31% and 98.53% identity ( Figure 1E).

**Figure 5.**
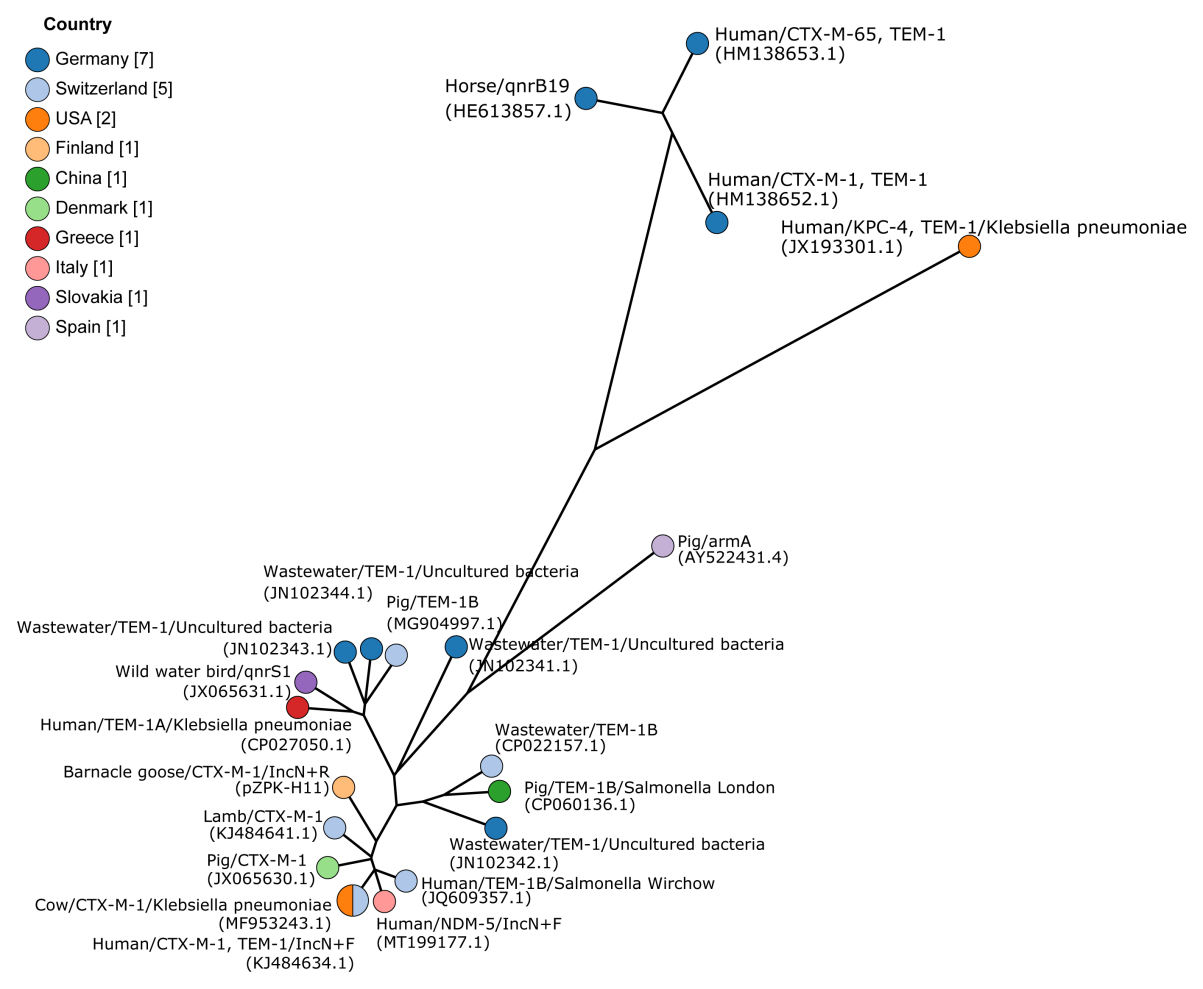
Minimum spanning tree based on core genome multilocus sequence typing of IncN+IncR multireplicon. Plasmid pZPK-H11 compared with 20 previously published plasmids from GenBank. Nodes are colored by the country of origin and node text includes information on the source, *bla* gene, or other resistance gene if the *bla* gene was not present, bacterial species if other than *Escherichia coli*, and replicon type if other than IncN. GenBank accession numbers are provided in parentheses.

The multireplicon plasmid harbored *bla*_CTX-M-1_ and additional resistance genes *qnrS1*, *aadA2b*, and *lnu(F)*. No virulence genes were detected on the multireplicon plasmid. The IncN replicon in the plasmid was identified as ST1 with pMLST analysis. Top two hits from a BLASTn search against the NCBI database were IncF+IncN type multireplicons both isolated from *E. coli* of human origin, one in Italy with *bla*_NDM-5_ (GenBank accession: MT199177.1; 60% coverage and 99.97% identity) and the other in Switzerland carrying *bla*_CTX-M-1_ (GenBank accession: KJ484634.1; 59% coverage and 99.97% identity) ( Table 1). Other top hits with BLASTn included IncN plasmids isolated from various sources, such as uncultured bacteria from wastewater treatment plant effluent in Germany, carrying *bla*_TEM-1_ (GenBank accessions: JN102342.1, JN102344.1, and JN102343.1). A cgMLST-based minimum spanning tree was built with 34 discriminatory loci (95% occurrence) and is visualized in Figure 5.

**Figure 6.**
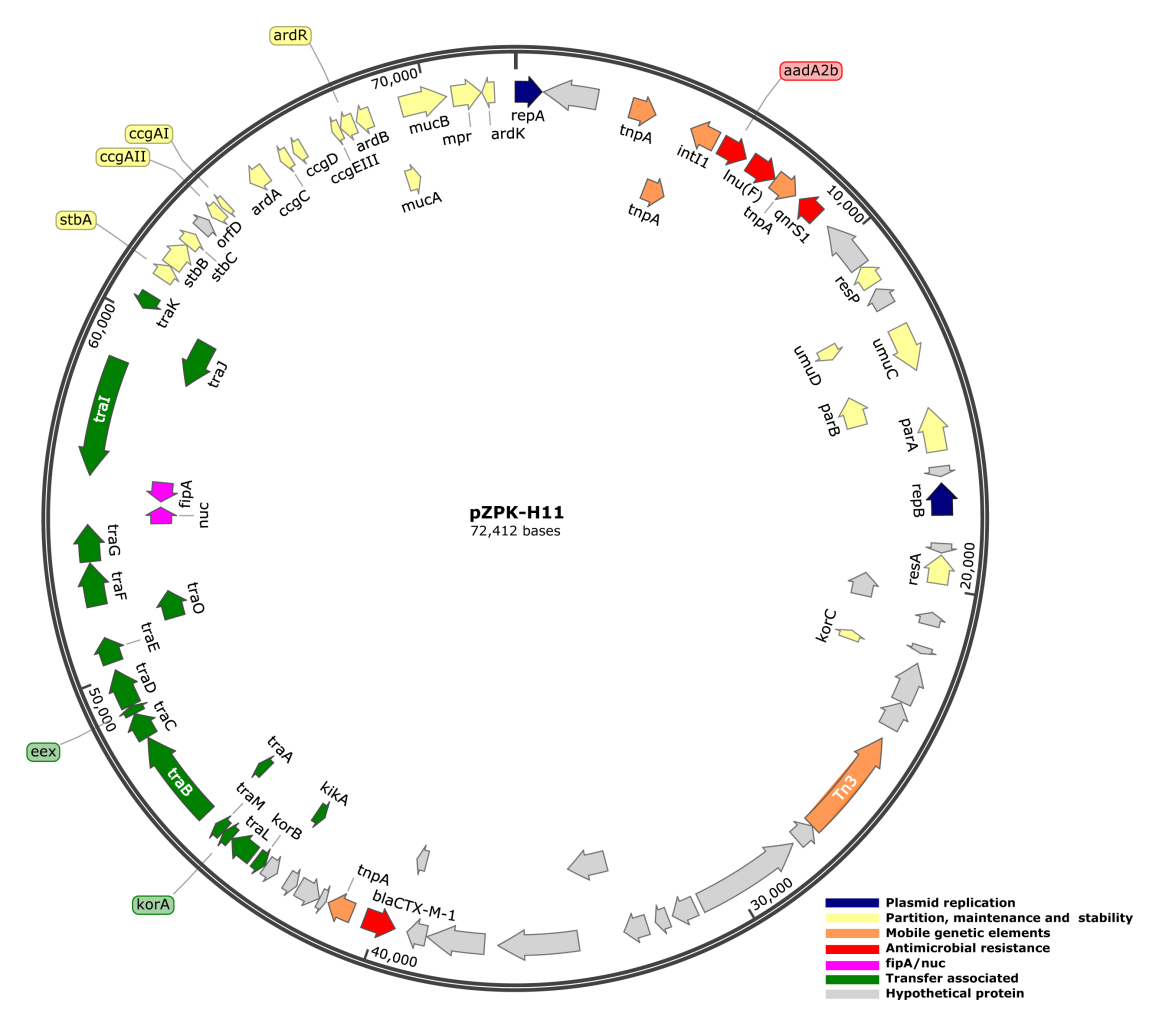
Genetic structure of multireplicon plasmid pZPK-H11. Predicted coding sequences and their orientation are represented by arrows colored based on the function of the gene product. Size of the plasmid is depicted on the outer circle.

pPK-H11 shared similar backbone structures with IncN reference R46, including the replicon *repA*, stability-related *stbABC*, *mucA*, and *mucB* involved in mutagenesis enhancement, genes involved in plasmid DNA protection from type I restriction enzymes ( *ccgC*, *ccgD*, *ccgAI*, and *ccgAII*), antirestriction-associated *ardA*, *ardB*, and *ardR*, and two transfer-associated *tra* loci ( *traI*, *traJ*, *traK*; *traL*, *traM*, *traA*, *traB*, *traC*, *traD*, *traE*, *traO*, *traF*, *traG*) ( Carattoli *et al.*, 2010; Delver & Belogurov, 1997; Dolejska *et al.*, 2013). Similar to the R46 structure, *fipA* and *nuc* were located between *tra* loci in pZPK-H11. IncR plasmid backbone structures identified in pZPK-H11 included replicon *repB*, *parAB*, and *umuCD* and multimer resolvase, but toxin–antitoxin *vagDC* operon involved in plasmid maintenance and *retA* reverse transcriptase were not identified ( Guo *et al.*, 2016). Transcriptional regulator *korC* was identified in a region not aligning with either IncN or IncR reference plasmids ( Ludwiczak *et al.*, 2013). *bla*_CTX-M-1_ was located next to mobile genetic element *tnpA* outside of a multidrug resistance cassette containing *aadA2b*, *lnu(F)*, and *qnrS1*, which were located downstream of *repA*. The pZPK-H11 structure is visualized in Figure 6.

## Discussion

The purpose of this study was to identify ESBL/pAmpC-producing *E. coli* in migrating barnacle geese residing near humans and to investigate plasmids harboring *bla* genes. The *bla* genes were encoded on four different plasmid replicons from nine different samples: IncI1 and IncK, the rare IncY, and the multireplicon IncN+IncR.

Interestingly, IncY harboring *bla*_CTX-M-15_ was found from two sequenced isolates with the same bacterial ST type (ST453) and AMR gene profile, indicating clonal spread of the bacteria between the animals or a common source. Plasmids of the IncY group are considered rarely detected phage-like plasmids with low copy numbers ( Meyer *et al.*, 1986; Rozwandowicz *et al.*, 2018). ESBL-producing *E. coli* harboring IncY have been detected in clinical human isolates ( Yasir *et al.*, 2020), a wastewater treatment plant in China ( Jiang *et al.*, 2019), and environment and fish samples in Tanzania ( Moremi *et al.*, 2016). The aforementioned human-associated finding of IncY raises the question of the origin of IncY in the barnacle geese samples. It can be speculated that the origin of AMR in geese is from anthropogenic sources.

Notable is the finding of the pZPK-H11 multireplicon IncN+IncR harboring *bla*_CTX-M-1_ in one of the sequenced isolates. IncN has been commonly found in animal fecal microbiota ( Carattoli, 2009) and has been identified as a disseminator of *bla*_CTX-M-1_ among animals and humans in Europe ( Dolejska *et al.*, 2013). *bla*_CTX-M-1_ has often been recovered from IncN ST1 ( Rozwandowicz *et al.*, 2018). In addition to *bla*_CTX-M-1_, the IncN+IncR multireplicon pZPK-H11 harbored resistance genes *aadA2b*, *lnu(F)*, and *qnrS1*. *qnr* genes have been previously associated with IncN plasmids from different *Salmonella* serovars isolated from human and poultry samples in the Netherlands ( García-Fernández *et al.*, 2009). It has been shown previously that IncR plasmids are able to form multireplicons with IncN, IncA/C, IncHI, and IncF type plasmids ( Drieux *et al.*, 2013; Jing *et al.*, 2019; Papagiannitsis *et al.*, 2013; Qu *et al.*, 2019). Previous studies indicate that IncR plasmids lack genes involved in conjugation ( Bielak *et al.*, 2011; Chen *et al.*, 2006), which indicates that pZPK-H11 is a putatively conjugative plasmid contributing to the transfer-associated genes located on the IncN region of the plasmid.

IncI1 carrying *bla*_CTX-M-1_ was identified in one of the isolates in our study. The finding of two identical IncI1 plasmids harboring *bla*_CMY-2_ from two samples, pZPK-H163 and pZPK-H193, indicates very recent clonal transmission within the flock or the possibility of samples originating from the same goose individual. IncK type plasmids with *bla*_CMY-2_ were recovered from two isolates in our study. IncK type plasmids carrying *bla*_CMY-2_ and *bla*_CTX-M-14_ have been previously isolated mainly from *E. coli* from animal sources in Europe ( Rozwandowicz *et al.*, 2018). The most prevalent resistance genes in the isolates in our study were AmpC type *bla*_CMY-2_ followed by *bla*_CTX-M-1_ and *bla*_CTX-M-15_. The finding of *bla*_CMY-2_ and *bla*_CTX-M-1_ on IncI1 plasmids is a common finding among poultry ( Accogli *et al.*, 2013; Leverstein-van Hall *et al.*, 2011), and these genes and plasmid replicons have been identified previously also from other wild avian species, such as seagulls and pelicans in Florida ( Poirel *et al.*, 2012). Wild birds of different species in Catalonia have also been found to harbor *bla*_CTX-M-15_ and *bla*_CMY-2_ among other beta-lactamases, such as *bla*_OXA-48_ in a barn owl ( Darwich *et al.*, 2019). No carbapenemases were recovered in our study. In addition, in a study by Bonnedahl *et al.* (2009), 9.4% of wild yellow-legged gulls in the south of France were found to harbor ESBL-producing *E. coli* and 6% more specifically *bla*_CTX-M-1_. The finding of beta-lactamases, especially in wild birds habituating aquatic environments, is worrisome because of the potential of bacterial transmission between humans and animals via surface water. Wild gulls in Sweden were found to harbor *bla*_CTX-M-15_/ *bla*_CTX-M-14_-producing *E. coli* similar to human and surface water isolates ( Atterby *et al.*, 2017). Wild birds may acquire resistance genes from anthropogenic sources and circulate resistance determinants again back into the human population, although contact with wild birds has been estimated to contribute only 0.3% of community-acquired intestinal carriage of ESBL/pAmpC-producing *E. coli* in humans in the Netherlands ( Mughini-Gras *et al.*, 2019). In addition to human health, wild migratory birds may play a role as disseminators of human-derived AMR to remote areas, potentially endangering the welfare of native animal species ( Hernández & González-Acuña, 2016).

Interestingly, the finding of ESBL/AmpC-producing *E. coli* in 4.5% of the studied barnacle geese fecal samples is slightly less than the prevalence of ESBL/AmpC-producing *E. coli* and *Klebsiella pneumoniae* described in Finnish asymptomatic human carriers (6.3%) ( Rintala *et al.*, 2018). AMR has been studied in wild birds previously, but this was the first time it was studied in migratory birds in Finland, and with WGS applied. The prevalence of 4.5% is relatively low compared with other studies conducted with different avian species. In a study conducted in the Netherlands ( Veldman *et al.*, 2013), ESBL/AmpC-producing *E. coli* were found in 15.7% of 21 different bird species tested, mainly originating from aquatic-associated species. In Spain, 15% of sampled birds were found to be positive for ESBL/pAmpC-producing *E. coli* ( Alcalá *et al.*, 2016), whereas in birds of prey in Germany and Mongolia the prevalence was found to be 13.8% and 10.8%, respectively ( Guenther *et al.*, 2012). The lower percentage in the present study could be partly explained by the different bird species examined. Barnacle geese do not feed on landfills or other human-related waste areas like gulls in certain areas ( Bonnedahl *et al.*, 2014) or other bird or mammal species like birds of prey but, as a waterfowl, may come into contact with human wastewater. Although the prevalence of ESBL/AmpC-producing *E. coli* in our study was less than in some other birds studied earlier, barnacle geese that have habituated to densely human-populated areas, such as recreational parks and public beaches ( Simões *et al.*, 2010), pose the potential risk of AMR spread to humans. Parks and public beaches can quickly become contaminated with fecal matter while flocks of hundreds of birds feed and defecate in the area. Additionally, household pets and children may come into contact with fecal matter more easily.

It has to be taken into account, however, that the sample size was limited. There have been studies of the effect of ultraviolet light on *E. coli* survival ( Vermeulen *et al.*, 2008; Whitman *et al.*, 2004), but to assess the effect of sunlight and other environmental factors on *E. coli* or resistance genes and plasmid survival in bird feces, more studies would need to be conducted. Seven out of nine samples positive for ESBL/AmpC-producing *E. coli* originated from samples collected in fall 2017, and temporal changes have been identified in ESBL-producing Enterobacteriaceae previously in urban Swedish mallards, with higher prevalence during warm summer months ( Hessman *et al.*, 2018).

To assess the actual risk of AMR in wildlife, bacteria, resistance genes, and mobile genetic elements have to be typed to a level where comparison between different sources, i.e., humans and animals, is plausible. WGS provides a powerful tool for studying phylogeny between different bacterial and plasmid reservoirs, and also enables the early detection of new resistance genes or successful plasmids. Long-read sequencing of the isolates allows for even more in-depth analysis of the location of resistance genes on plasmid replicons.

As *E. coli* belong to the normal intestinal microbiota of animals and virulence potential varies between strains, the public health relevance of ESBL/AmpC-producing *E. coli* derived from barnacle geese feces should be interpreted with caution. By examining virulence traits, however, it is possible to identify potential pathogens. The increased serum survival gene *iss* was found from all of the sequenced isolates. *Iss* has been identified in extraintestinal pathogenic *E. coli* (ExPEC) as well as in avian pathogenic *E. coli* (APEC) ( Johnson *et al.*, 2008; Tivendale *et al.*, 2004). *Iss* and *iroN* have been found to be prevalent in *E. coli*, causing salpingitis in egg-laying hens ( Poulsen *et al.*, 2020), and have also been identified in *E. coli* in poultry production ( Kim *et al.*, 2020; Oikarainen *et al.*, 2019). The glutamate decarboxylase gene *gad* was also prevalent in the sequenced isolates, and it has been identified frequently in APEC in broilers together with *iss* and *iroN* ( Azam *et al.*, 2020). These findings highlight the potential of AMR transmission via virulent *E. coli* strains between bird species, as well as between other animals and potentially humans.

Wildlife may serve as a reservoir for resistant bacteria ( Carroll *et al.*, 2015) and more than 60% of emerging infectious diseases since the 1940s have been zoonotic ( Jones *et al.*, 2008), which makes the continuous surveillance of wildlife pathogens extremely important. Our study highlights the fact that wild birds living in close contact with humans carry bacteria with plasmid-mediated beta-lactamases and may pose a potential risk of bacterial clonal spread or horizontal gene transfer through the environment.

## Conclusion

Wild migratory geese were found to carry ESBL/AmpC-producing *E. coli* with diverse bacterial STs and the assessment of samples with WGS revealed the location of resistance genes in specific plasmid replicons. A novel multireplicon, IncN+IncR, carrying *bla*_CTX-M-1_ was recovered, and human-associated *bla*_CTX-M-15_ was found from two IncY plasmids. The findings indicate that wildlife carrying plasmids and resistance genes are potentially worrisome for public health. In addition to successful, more frequently identified plasmid replicons IncI1, IncK, and IncN, the rare IncY plasmid and IncN+IncR multireplicon plasmids were recovered in our study, indicating that wildlife carry epidemic plasmids and also serve as potential disseminators of plasmids currently viewed as uncommon in humans and livestock. Although the prevalence of ESBL/AmpC-producing *E. coli* was relatively moderate at 4.5%, the transmission potential should not be underestimated, especially in urban areas with dense human and animal populations. Continuous and prudent monitoring of resistance determinants in different environments is vital for understanding evolving resistance patterns and the epidemiology of AMR within and between the different One Health compartments.

## Data availability

EMBL-EBI European Nucleotide Archive: Raw reads and plasmid sequences. Accession number PRJEB42655; https://identifiers.org/ena.embl:PRJEB42655.

Accession numbers for each isolate are provided in Table 2.

## References

[ref-1] Accogli M Fortini D Giufrè M : IncI1 plasmids associated with the spread of CMY-2, CTX-M-1 and SHV-12 in *Escherichia coli* of animal and human origin. *Clin Microbiol Infect.* 2013;19(5):E238–E240. 10.1111/1469-0691.12128 23331857

[ref-2] Alcalá L Alonso CA Simón C : Wild Birds, Frequent Carriers of Extended-Spectrum β-Lactamase (ESBL) Producing *Escherichia coli* of CTX-M and SHV-12 Types. *Microb Ecol.* 2016;72(4):861–869. 10.1007/s00248-015-0718-0 26687342

[ref-3] Alikhan NF Petty NK Ben Zakour NL : BLAST Ring Image Generator (BRIG): Simple prokaryote genome comparisons. *BMC Genomics.* 2011;12:402. 10.1186/1471-2164-12-402 21824423 PMC3163573

[ref-4] Atterby C Börjesson S Ny S : ESBL-producing *Escherichia coli* in Swedish gulls—A case of environmental pollution from humans? *PLoS One.* 2017;12(12):e0190380. 10.1371/journal.pone.0190380 29284053 PMC5746268

[ref-5] Azam M Mohsin M Johnson TJ : Genomic landscape of multi-drug resistant avian pathogenic *Escherichia coli* recovered from broilers. *Vet Microbiol.* 2020;247:108766. 10.1016/j.vetmic.2020.108766 32768218

[ref-6] Bielak E Bergenholtz RD Jørgensen MS : Investigation of diversity of plasmids carrying the bla _TEM-52_ gene. *J Antimicrob Chemother.* 2011;66(11):2465–2474. 10.1093/jac/dkr331 21831988

[ref-7] Bonnedahl J Drobni M Gauthier-Clerc M : Dissemination of *Escherichia coli* with CTX-M type ESBL between humans and yellow-legged gulls in the south of France. *PLoS One.* 2009;4(6):e5958. 10.1371/journal.pone.0005958 19536298 PMC2694269

[ref-8] Bonnedahl J Hernandez J Stedt J : Extended-spectrum β-lactamases in *Escherichia coli* and *Klebsiella pneumoniae* in Gulls, Alaska, USA. *Emerg Infect Dis.* 2014;20(5):897–899. 10.3201/eid2005.130325 24750592 PMC4012786

[ref-9] Bonnedahl J Järhult JD : Antibiotic resistance in wild birds. *Ups J Med Sci.* 2014;119(2):113–116. 10.3109/03009734.2014.905663 24697355 PMC4034547

[ref-10] Brouwer MSM Tagg KA Mevius DJ : IncI shufflons: Assembly issues in the next-generation sequencing era. *Plasmid.* 2015;80:111–117. 10.1016/j.plasmid.2015.04.009 25952328

[ref-11] Carattoli A : Resistance plasmid families in *Enterobacteriaceae*. *Antimicrob Agents Chemother.* 2009;53(6):2227–2238. 10.1128/AAC.01707-08 19307361 PMC2687249

[ref-12] Carattoli A Aschbacher R March A : Complete nucleotide sequence of the IncN plasmid pKOX105 encoding VIM-1, QnrS1 and SHV-12 proteins in Enterobacteriaceae from Bolzano, Italy compared with IncN plasmids encoding KPC enzymes in the USA. *J Antimicrob Chemother.* 2010;65(10):2070–2075. 10.1093/jac/dkq269 20656680

[ref-13] Carattoli A : Plasmids in Gram negatives: Molecular typing of resistance plasmids. *Int J Med Microbiol.* 2011;301(8):654–658. 10.1016/j.ijmm.2011.09.003 21992746

[ref-14] Carattoli A : Plasmids and the spread of resistance. *Int J Med Microbiol.* 2013;303(6–7):298–304. 10.1016/j.ijmm.2013.02.001 23499304

[ref-15] Carattoli A Zankari E Garciá-Fernández A : *In Silico* detection and typing of plasmids using plasmidfinder and plasmid multilocus sequence typing. *Antimicrob Agents Chemother.* 2014;58(7):3895–3903. 10.1128/AAC.02412-14 24777092 PMC4068535

[ref-16] Carroll D Wang J Fanning S : Antimicrobial Resistance in Wildlife: Implications for Public Health. *Zoonoses Public Health.* 2015;62(7):534–542. 10.1111/zph.12182 25639901

[ref-17] CDC (Centers for Disease Control and Prevention): Antibiotic resistance threats in the United States, 2019.Atlanta, GA; U.S. Department of Health and Human Services, CDC. 2019. 10.15620/cdc:82532

[ref-18] Chen YT Shu HY Li LH : Complete nucleotide sequence of pK245, a 98-kilobase plasmid conferring quinolone resistance and extended-spectrum-beta-lactamase activity in a clinical *Klebsiella pneumoniae* isolate. *Antimicrob Agents Chemother.* 2006;50(11):3861–3866. 10.1128/AAC.00456-06 16940067 PMC1635178

[ref-19] Clausen PTLC Aarestrup FM Lund O : Rapid and precise alignment of raw reads against redundant databases with KMA. *BMC Bioinformatics.* 2018;19(1):307. 10.1186/s12859-018-2336-6 30157759 PMC6116485

[ref-20] Darwich L Vidal A Seminati C : High prevalence and diversity of extended-spectrum β-lactamase and emergence of OXA-48 producing *Enterobacterales* in wildlife in Catalonia. *PLoS One.* 2019;14(8):e0210686. 10.1371/journal.pone.0210686 31381578 PMC6681944

[ref-21] Day MJ Hopkins KL Wareham DW : Extended-spectrum β-lactamase-producing *Escherichia coli* in human-derived and foodchain-derived samples from England, Wales, and Scotland: an epidemiological surveillance and typing study. *Lancet Infect Dis.* 2019;19(12):1325–1335. 10.1016/S1473-3099(19)30273-7 31653524

[ref-22] Delver EP Belogurov AA : Organization of the leading region of IncN plasmid pKM101 (R46): A regulation controlled by CUP sequence elements. *J Mol Biol.* 1997;271(1):13–30. 10.1006/jmbi.1997.1124 9300052

[ref-23] Drieux L Decré D Frangeul L : Complete nucleotide sequence of the large conjugative pTC2 multireplicon plasmid encoding the VIM-1 metallo-β-lactamase. *J Antimicrob Chemother.* 2013;68(1):97–100. 10.1093/jac/dks367 23011287

[ref-24] Dolejska M Villa L Hasman H : Characterization of IncN plasmids carrying bla *CTX-M-1* and qnr genes in *Escherichia coli* and Salmonella from animals, the environment and humans. *J Antimicrob Chemother.* 2013;68(2):333–339. 10.1093/jac/dks387 23060365

[ref-25] Dolejska M Papagiannitsis CC : Plasmid-mediated resistance is going wild. *Plasmid.* 2018;99:99–111. 10.1016/j.plasmid.2018.09.010 30243983

[ref-26] EFSA (European Food Safety Authority): Scientific Opinion on the public health risks of bacterial strains producing extended-spectrum β-lactamases and/or AmpC β-lactamases in food and food-producing animals. *EFSA J.* 2011;9(8):2322. 10.2903/j.efsa.2011.2322

[ref-27] EFSA (European Food Safety Authority) and ECDC (European Centre for Disease Prevention and Control): The European Union Summary Report on Antimicrobial Resistance in zoonotic and indicator bacteria from humans, animals and food in 2017/2018. *EFSA J.* 2020;18(3):6007. 10.2903/j.efsa.2020.6007 32874244 PMC7448042

[ref-28] Elmberg J Berg C Lerner H : Potential disease transmission from wild geese and swans to livestock, poultry and humans: a review of the scientific literature from a One Health perspective. *Infect Ecol Epidemiol.* 2017;7(1):1300450. 10.1080/20008686.2017.1300450 28567210 PMC5443079

[ref-29] EUCAST: European Committee on Antimicrobial Susceptibility Testing. EUCAST Disk Diffusion Method Manual 6.0.2017; [Accessed December 19, 2018]. Reference Source

[ref-30] EUCAST: European Committee on Antimicrobial Susceptibility Testing. Antimicrobial wild type, distributions of microorganisms.2019; [Accessed January 27, 2019]. Reference Source

[ref-31] Ewers C Bethe A Semmler T : Extended-spectrum β-lactamase-producing and AmpC-producing Escherichia *coli* from livestock and companion animals, and their putative impact on public health: A global perspective. *Clin Microbiol Infect.* 2012;18(7):646–655. 10.1111/j.1469-0691.2012.03850.x 22519858

[ref-32] Fox AD Leafloor JO : A global audit of the status and trends of Arctic and Northern Hemisphere goose populations. Conservation of Arctic Flore and Fauna International Secretariat: Akureyri, Iceland.2018. Reference Source

[ref-33] Frankel G Phillips AD Rosenshine I : Enteropathogenic and enterohaemorrhagic *Escherichia coli* : more subversive elements. *Mol Microbiol.* 1998;30(5):911–921. 10.1046/j.1365-2958.1998.01144.x 9988469

[ref-34] García-Fernández A Fortini D Veldman K : Characterization of plasmids harbouring *qnrS1*, *qnrB2* and *qnrB19* genes in *Salmonella*. *J Antimicrob Chemother.* 2009;63(2):274–281. 10.1093/jac/dkn470 19001452

[ref-35] Guenther S Aschenbrenner K Stamm I : Comparable High Rates of Extended-Spectrum-Beta-Lactamase-Producing *Escherichia coli* in Birds of Prey from Germany and Mongolia. *PLoS One.* 2012;7(12):e53039. 10.1371/journal.pone.0053039 23300857 PMC3534101

[ref-36] Guo Q Spychala CN McElheny CL : Comparative analysis of an IncR plasmid carrying *armA*, *bla* _DHA-1_ and *qnrB4* from *Klebsiella pneumoniae* ST37 isolates. *J Antimicrob Chemother.* 2016;71(4):882–886. 10.1093/jac/dkv444 26747096 PMC4790621

[ref-37] Hasman H Saputra D Sicheritz-Ponten T : Rapid whole-genome sequencing for detection and characterization of microorganisms directly from clinical samples. *J Clin Microbiol.* 2014;52(1):139–146. 10.1128/JCM.02452-13 24172157 PMC3911411

[ref-38] Hatha AAM Divya PS Saramma AV : Migratory bird, Branta leucopis (Barnacle goose), a potential carrier of diverse Escherichia coli serotypes into pristime Arctic environment. *Curr Sci.* 2013;104(8):1078–1080. [Accessed June 24, 2020]. Reference Source

[ref-39] Hernández J González-Acuña D : Anthropogenic antibiotic resistance genes mobilization to the polar regions. *Infect Ecol Epidemiol.* 2016;6:32112. 10.3402/iee.v6.32112 27938628 PMC5149653

[ref-40] Hessman J Atterby C Olsen B : High Prevalence and Temporal Variation of Extended Spectrum β-Lactamase-Producing Bacteria in Urban Swedish Mallards. *Microb Drug Resist.* 2018;24(6):822–829. 10.1089/mdr.2017.0263 29304312

[ref-41] Holmes AH Moore LSP Sundsfjord A : Understanding the mechanisms and drivers of antimicrobial resistance. *Lancet.* 2016;387(10014):176–187. 10.1016/S0140-6736(15)00473-0 26603922

[ref-42] Jensen GH Madsen J Nagy S : AEWA International Single Species Management Plan for the Barnacle Goose (Branta leucopsis) - Russia/Germany & Netherlands population, East Greenland/Scotland & Ireland population, Svalbard/South-west Scotland population. AEWA Technical Series No. 70. Bonn, Germany.2018. Reference Source

[ref-43] Jiang X Cui X Xu H : Whole Genome Sequencing of Extended-Spectrum Beta-Lactamase (ESBL)-Producing *Escherichia coli* Isolated From a Wastewater Treatment Plant in China. *Front Microbiol.* 2019;10:1797. 10.3389/fmicb.2019.01797 31428078 PMC6688389

[ref-44] Jing Y Jiang X Yin Z : Genomic diversification of IncR plasmids from China. *J Glob Antimicrob Resist.* 2019;19:358–364. 10.1016/j.jgar.2019.06.007 31216492

[ref-45] Joensen KG Scheutz F Lund O : Real-time whole-genome sequencing for routine typing, surveillance, and outbreak detection of verotoxigenic *Escherichia coli*. *J Clin Microbiol.* 2014;52(5):1501–10. 10.1128/JCM.03617-13 24574290 PMC3993690

[ref-46] Johnson TJ Wannemuehler YM Nolan LK : Evolution of the *iss* gene in *Escherichia coli*. *Appl Environ Microbiol.* 2008;74(8):2360–2369. 10.1128/AEM.02634-07 18281426 PMC2293169

[ref-47] Jones KE Patel NG Levy MA : Global trends in emerging infectious diseases. *Nature.* 2008;451(7181):990–993. 10.1038/nature06536 18288193 PMC5960580

[ref-48] Kim YB Yoon MY Ha JS : Molecular characterization of avian pathogenic *Escherichia coli* from broiler chickens with colibacillosis. *Poult Sci.* 2020;99(2):1088–1095. 10.1016/j.psj.2019.10.047 32029145 PMC7587703

[ref-49] Kuczkowski M Krawiec M Voslamber B : Virulence Genes and the Antimicrobial Susceptibility of *Escherichia coli*, Isolated from Wild Waterbirds, in the Netherlands and Poland. *Vector Borne Zoonotic Dis.* 2016;16(8):528–536. 10.1089/vbz.2015.1935 27348207

[ref-56] Lääveri T Vlot JA van Dam AP : Extended-spectrum beta-lactamase-producing *Enterobacteriaceae* (ESBL-PE) among travellers to Africa: Destination-specific data pooled from three European prospective studies. *BMC Infect Dis.* 2018;18(1):341. 10.1186/s12879-018-3245-z 30037325 PMC6057027

[ref-50] Larsen MV Cosentino S Rasmussen S : Multilocus sequence typing of total-genome-sequenced bacteria. *J Clin Microbiol.* 2012;50(4):1355–61. 10.1128/JCM.06094-11 22238442 PMC3318499

[ref-51] Larsen MV Cosentino S Lukjancenko O : Benchmarking of methods for genomic taxonomy. *J Clin Microbiol.* 2014;52(5):1529–39. 10.1128/JCM.02981-13 24574292 PMC3993634

[ref-52] Leverstein-van Hall MA Dierikx CM Stuart JC : Dutch patients, retail chicken meat and poultry share the same ESBL genes, plasmids and strains. *Clin Microbiol Infect.* 2011;17(6):873–880. 10.1111/j.1469-0691.2011.03497.x 21463397

[ref-53] Liu YY Lin JW Chen CC : cano-wgMLST_BacCompare: A Bacterial Genome Analysis Platform for Epidemiological Investigation and Comparative Genomic Analysis. *Front Microbiol.* 2019;10:1687. 10.3389/fmicb.2019.01687 31396192 PMC6668299

[ref-54] Llarena AK Skarp-de Haan CPA Rossi M : Characterization of the *Campylobacter jejuni* Population in the Barnacle Geese Reservoir. *Zoonoses Public Health.* 2015;62(3):209–221. 10.1111/zph.12141 24948379

[ref-55] Ludwiczak M Dolowy P Markowska A : Global transcriptional regulator KorC coordinates expression of three backbone modules of the broad-host-range RA3 plasmid from IncU incompatibility group. *Plasmid.* 2013;70(1):131–145. 10.1016/j.plasmid.2013.03.007 23583562

[ref-57] Meyer J Stålhammar-Carlemalm M Streiff M : Sequence relations among the IncY plasmid p15B, P1, and P7 prophages. *Plasmid.* 1986;16(2):81–89. 10.1016/0147-619x(86)90066-1 3749335

[ref-58] Moremi N Manda EV Falgenhauer L : Predominance of CTX-M-15 among ESBL producers from environment and fish gut from the shores of Lake Victoria in Mwanza, Tanzania. *Front Microbiol.* 2016;7:1862. 10.3389/fmicb.2016.01862 27990135 PMC5130978

[ref-59] Mughini-Gras L Dorado-García A van Duijkeren E : Attributable sources of community-acquired carriage of *Escherichia coli* containing β-lactam antibiotic resistance genes: a population-based modelling study. *Lancet Planet Health.* 2019;3(8):e357–e369. 10.1016/S2542-5196(19)30130-5 31439317

[ref-60] Müller A Stephan R Nüesch-Inderbinen M : Distribution of virulence factors in ESBL-producing *Escherichia coli* isolated from the environment, livestock, food and humans. *Sci Total Environ.* 2016;541:667–672. 10.1016/j.scitotenv.2015.09.135 26437344

[ref-61] Niskanen T Waldenström J Fredriksson-Ahomaa M : *virF*-positive *Yersinia pseudotuberculosis* and *Yersinia enterocolitica* found in migratory birds in Sweden. *Appl Environ Microbiol.* 2003;69(8):4670–4675. 10.1128/aem.69.8.4670-4675.2003 12902256 PMC169077

[ref-62] Nordmann P Poirel L : Epidemiology and Diagnostics of Carbapenem Resistance in Gram-negative Bacteria. *Clin Infect Dis.* 2019;69(Suppl 7):S521–S528. 10.1093/cid/ciz824 31724045 PMC6853758

[ref-63] Oikarainen PE Pohjola LK Pietola ES : Direct vertical transmission of ESBL/pAmpC-producing *Escherichia coli* limited in poultry production pyramid. *Vet Microbiol.* 2019;231:100–106. 10.1016/j.vetmic.2019.03.001 30955795

[ref-64] Papagiannitsis CC Miriagou V Giakkoupi P : Characterization of pKP1780, a novel IncR plasmid from the emerging *Klebsiella pneumoniae* ST147, encoding the VIM-1 metallo-Β-lactamase. *J Antimicrob Chemother.* 2013;68(10):2259–2262. 10.1093/jac/dkt196 23711894

[ref-65] Poirel L Potron A De La Cuesta C : Wild coastline birds as reservoirs of broad-spectrum-β-lactamase-producing *Enterobacteriaceae* in Miami Beach, Florida. *Antimicrob Agents Chemother.* 2012;56(5):2756–2758. 10.1128/AAC.05982-11 22314536 PMC3346599

[ref-66] Poulsen LL Kudirkiene E Jørgensen SL : Whole genome sequence comparison of avian pathogenic *Escherichia coli* from acute and chronic salpingitis of egg laying hens. *BMC Vet Res.* 2020;16(1):148. 10.1186/s12917-020-02369-5 32434525 PMC7238577

[ref-67] Qu D Shen Y Hu L : Comparative analysis of KPC-2-encoding chimera plasmids with multi-replicon IncR:Inc _pA1763-KPC_:IncN1 or IncFII _pHN7A8_:Inc _pA1763-KPC_:IncN1. *Infect Drug Resist.* 2019;12:285–296. 10.2147/IDR.S189168 30774396 PMC6353027

[ref-68] Rintala ESA Gröndahl-Yli-Hannuksela K Lönnqvist E : ESBL:ää tuottavien suolistobakteerien oireeton kantajuus Etelä-Suomessa.Suom Lääril 43/2018;73:2503-2513.2018. Reference Source

[ref-69] Rozwandowicz M Brouwer MSM Fischer J : Plasmids carrying antimicrobial resistance genes in Enterobacteriaceae. *J Antimicrob Chemother.* 2018;73(5):1121–1137. 10.1093/jac/dkx488 29370371

[ref-70] Sandegren L Stedt J Lustig U : Long-term carriage and rapid transmission of extended spectrum beta-lactamase-producing *E. coli* within a flock of Mallards in the absence of antibiotic selection. *Environ Microbiol Rep.* 2018;10(5):576–582. 10.1111/1758-2229.12681 30043488

[ref-71] Seemann T : Prokka: rapid prokaryotic genome annotation. *Bioinformatics.* 2014;30(14):2068–2069. 10.1093/bioinformatics/btu153 24642063

[ref-72] Simões RR Poirel L Da Costa PM : Seagulls and beaches as reservoirs for multidrug-resistant *Escherichia coli*. *Emerg Infect Dis.* 2010;16(1):110–112. 10.3201/eid1601.090896 20031053 PMC2874366

[ref-73] Stedt J Bonnedahl J Hernandez J : Carriage of CTX-M type extended spectrum β-lactamases (ESBLs) in gulls across Europe. *Acta Vet Scand.* 2015;57:74. 10.1186/s13028-015-0166-3 26526188 PMC4629291

[ref-74] SYKE (Suomen ympäristökeskus) > Ymparisto >: Valkoposkihanhien seuranta.Published 9.9. 2016, updated 23.9. 2019. [Accessed June 23, 2020]. Reference Source

[ref-75] SYKE (Suomen ympäristökeskus) >: Valkoposkihanhien väheneminen pääkaupunkiseudulla taittui.Published 7.8.2019. [Accessed June 23, 2020]. Reference Source

[ref-76] Tivendale KA Allen JL Ginns CA : Association of iss and iucA, but not tsh, with plasmid-mediated virulence of avian pathogenic *Escherichia coli*. *Infect Immun.* 2004;72(11):6554–6560. 10.1128/IAI.72.11.6554-6560.2004 15501787 PMC523064

[ref-77] van der Bij AK Pitout JDD : The role of international travel in the worldwide spread of multiresistant Enterobacteriaceae. *J Antimicrob Chemother.* 2012;67(9):2090–2100. 10.1093/jac/dks214 22678728

[ref-78] Veldman K van Tulden P Kant A : Characteristics of cefotaxime-resistant *Escherichia coli* from wild birds in The Netherlands. *Appl Environ Microbiol.* 2013;79(24):7556–7561. 10.1128/AEM.01880-13 24038683 PMC3837810

[ref-79] Vermeulen N Keeler WJ Nandakumar K : The bactericidal effect of ultraviolet and visible light on *Escherichia coli*. *Biotechnol Bioeng.* 2008;99(3):550–556. 10.1002/bit.21611 17680675

[ref-80] Whitman RL Nevers MB Korinek GC : Solar and temporal effects on *Escherichia coli* concentration at a Lake Michigan swimming beach. *Appl Environ Microbiol.* 2004;70(7):4276–4285. 10.1128/AEM.70.7.4276-4285.2004 15240311 PMC444827

[ref-81] WHO Global Health Observatory. [Accessed June 22, 2020]. Reference Source

[ref-82] Wick RR Judd LM Gorrie CL : Unicycler: Resolving bacterial genome assemblies from short and long sequencing reads. *PLoS Comput Biol.* 2017;13(6):e1005595. 10.1371/journal.pcbi.1005595 28594827 PMC5481147

[ref-83] Wirth T Falush D Lan R : Sex and virulence in *Escherichia coli*: An evolutionary perspective. *Mol Microbiol.* 2006;60(5):1136–1151. 10.1111/j.1365-2958.2006.05172.x 16689791 PMC1557465

[ref-84] Woerther PL Andremont A Kantele A : Travel-acquired ESBL-producing *Enterobacteriaceae*: impact of colonization at individual and community level. *J Travel Med.* 2017;24(suppl_1):S29–S34. 10.1093/jtm/taw101 28520999 PMC5441303

[ref-85] Yasir M Farman M Shah MW : Genomic and antimicrobial resistance genes diversity in multidrug-resistant CTX-M-positive isolates of *Escherichia coli* at a health care facility in Jeddah. *J Infect Public Health.* 2020;13(1):94–100. 10.1016/j.jiph.2019.06.011 31279801

[ref-86] Yrjölä RA Holopainen S Pakarinen R : The Barnacle Goose ( *Branta leucopsis*) in the archipelago of southern Finland - population growth and nesting dispersal.2017. Reference Source

[ref-87] Zankari E Hasman H Cosentino S : Identification of acquired antimicrobial resistance genes. *J Antimicrob Chemother.* 2012;67(11):2640–4. 10.1093/jac/dks261 22782487 PMC3468078

[ref-88] Zhou Z Alikhan NF Sergeant MJ : Grapetree: Visualization of core genomic relationships among 100,000 bacterial pathogens. *Genome Res.* 2018;28(9):1395–1404. 10.1101/gr.232397.117 30049790 PMC6120633

