# Supplementary material for: Plasmids conferring resistance to extended-spectrum beta-lactamases including a rare IncN+IncR multireplicon carrying bla CTX-M-1 in Escherichia coli recovered from migrating barnacle geese ( Branta leucopsis)
Source: Open Res Eur. 2021 May 4;1:46. [Version 1] doi: 10.12688/openreseurope.13529.1 (PMC10446048; doi:10.12688/openreseurope.13529.1)
Supplement: Peer review history

# Peer review: Reviewer response for version 1

**DOI:** 10.21956/openreseurope.14599.r28186

**Published:** 2022-01-04

**Authors:** Magdalena Maria Zając

**Affiliations:** 1 Department of Microbiology, National Veterinary Research Institute, Puławy, Poland

The authors of manuscript titled "Plasmids conferring resistance to extended-spectrum beta-lactamases including a rare IncN+IncR multireplicon carrying blaCTX-M-1 in *Escherichia coli* recovered from migrating barnacle geese ( *Branta leucopsis*)” have presented interesting and well-described research which can have a wide audience. They proved that migratory geese carry ESBL/ampC producing *E. coli*  and identified and described in details plasmids harboring resistance genes by analyses of whole genome sequences.

 The manuscript is easy to read and clear. All manuscript sections are high quality writing, detailed description of used methods and tools allows to repeat the research. The sequences are easily accessible at NCBI database. I do not have any remarks.

Is the study design appropriate and does the work have academic merit?

Yes

Is the work clearly and accurately presented and does it cite the current literature?

Yes

If applicable, is the statistical analysis and its interpretation appropriate?

Not applicable

Are all the source data underlying the results available to ensure full reproducibility?

Yes

Are the conclusions drawn adequately supported by the results?

Yes

Are sufficient details of methods and analysis provided to allow replication by others?

Yes

Reviewer Expertise:

AMR in Salmonella and E. coli, whole genome sequencing analysis

I confirm that I have read this submission and believe that I have an appropriate level of expertise to confirm that it is of an acceptable scientific standard.

# Peer review: Reviewer response for version 1

**DOI:** 10.21956/openreseurope.14599.r26903

**Published:** 2021-06-08

**Authors:** Angela H. A. M. van Hoek

**Affiliations:** 1 Centre for Infectious Disease Control (CIb), National Institute for Public Health and the Environment (RIVM), Bilthoven, The Netherlands

The authors of "Plasmids conferring resistance to extended-spectrum beta-lactamases including a rare IncN+IncR multireplicon carrying *bla*_CTX-M-1_ in *Escherichia coli* recovered from migrating barnacle geese ( *Branta leucopsis*) have written a very clear and easy to read paper that is of interest to many readers in the current One Health and AMR era. The whole genome sequencing data is publicly available, which allows re-analysis of the results. I have only a few issues with the manuscript. 

 I find the term core genome MLST (cgMLST) while comparing various IncI1 type (figure 2), IncIK type (figure 3), incY type (figure 4) and IncN+IncR type (figure 5) plasmids confusing. The legends of these figures also miss the number of loci included in the analysis, one has to find this somewhere in the results sections. The number of allelic distances would be much appreciated in the minimum spanning trees of figures 2 - 4.

 The finding of EPEC isolates among the spring isolates should be described in more detail. Since so many tools were used from the Center for Genomic Epidemiology website (like MLST, ResFinder, PlasmidFinder) this reader/reviewer would also appreciate the SerotypeFinder results ending up in the manuscript.

Is the study design appropriate and does the work have academic merit?

Yes

Is the work clearly and accurately presented and does it cite the current literature?

Yes

If applicable, is the statistical analysis and its interpretation appropriate?

Yes

Are all the source data underlying the results available to ensure full reproducibility?

Yes

Are the conclusions drawn adequately supported by the results?

Yes

Are sufficient details of methods and analysis provided to allow replication by others?

Yes

Reviewer Expertise:

WGS analysis of AMR (pathogenic) zoonotic bacteria

I confirm that I have read this submission and believe that I have an appropriate level of expertise to confirm that it is of an acceptable scientific standard.
